# Genomic transcription factor binding site selection is edited by the chromatin remodeling factor CHD4

**DOI:** 10.1093/nar/gkae025

**Published:** 2024-01-28

**Authors:** Mika Saotome, Deepak B Poduval, Sara A Grimm, Aerica Nagornyuk, Sakuntha Gunarathna, Takashi Shimbo, Paul A Wade, Motoki Takaku

**Affiliations:** Department of Biomedical Sciences, University of North Dakota School of Medicine and Health Sciences, Grand Forks, ND 58202, USA; Department of Biomedical Sciences, University of North Dakota School of Medicine and Health Sciences, Grand Forks, ND 58202, USA; Biostatistics and Computational Biology Branch, National Institute of Environmental Health Sciences, Research Triangle Park, NC 27709, USA; Department of Biomedical Sciences, University of North Dakota School of Medicine and Health Sciences, Grand Forks, ND 58202, USA; Department of Biomedical Sciences, University of North Dakota School of Medicine and Health Sciences, Grand Forks, ND 58202, USA; Epigenetics and Stem Cell Biology Laboratory, National Institute of Environmental Health Sciences, Research Triangle Park, NC 27709, USA; Epigenetics and Stem Cell Biology Laboratory, National Institute of Environmental Health Sciences, Research Triangle Park, NC 27709, USA; Department of Biomedical Sciences, University of North Dakota School of Medicine and Health Sciences, Grand Forks, ND 58202, USA

## Abstract

Biologically precise enhancer licensing by lineage-determining transcription factors enables activation of transcripts appropriate to biological demand and prevents deleterious gene activation. This essential process is challenged by the millions of matches to most transcription factor binding motifs present in many eukaryotic genomes, leading to questions about how transcription factors achieve the exquisite specificity required. The importance of chromatin remodeling factors to enhancer activation is highlighted by their frequent mutation in developmental disorders and in cancer. Here, we determine the roles of CHD4 in enhancer licensing and maintenance in breast cancer cells and during cellular reprogramming. In unchallenged basal breast cancer cells, CHD4 modulates chromatin accessibility. Its depletion leads to redistribution of transcription factors to previously unoccupied sites. During cellular reprogramming induced by the pioneer factor GATA3, CHD4 activity is necessary to prevent inappropriate chromatin opening. Mechanistically, CHD4 promotes nucleosome positioning over GATA3 binding motifs to compete with transcription factor–DNA interaction. We propose that CHD4 acts as a chromatin proof-reading enzyme that prevents unnecessary gene expression by editing chromatin binding activities of transcription factors.

## Graphical Abstract

**Graphical Abstract:**
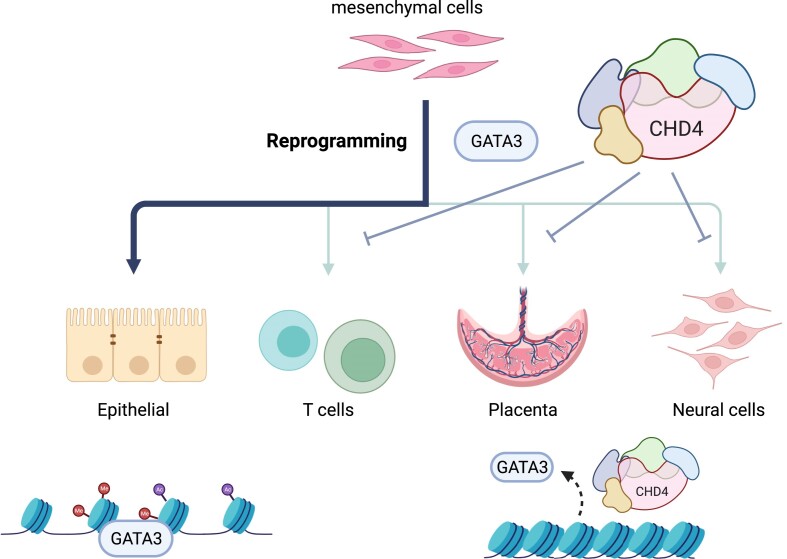

## Introduction

During the cell fate transitions integral to development or transcription factor-dependent cellular reprogramming, lineage-determining transcription factors (TFs) must contend with chromatin to nucleate active enhancers. Some transcription factors such as pioneer factors utilize their intrinsic ability to bind nucleosomal DNA in closed chromatin as a first step in the induction of chromatin opening (1,2). Establishment of an active enhancer is hypothesized to be a multi-step process involving alterations to local chromatin by chromatin remodeling enzymes, histone replacement, and editing of histone and DNA modification (3,4). All downstream processes flow from the initial event—recognition of DNA sequence by a sequence-specific DNA binding transcription factor. In most cases, TFs are present on the order of thousands to tens of thousands of molecules per cell (5,6). By contrast, many transcription factor motifs are present on the order of millions of copies per genome (7–9). How TFs are directed to activate enhancers in all the correct locations and only the correct locations is critical, as both failure to activate appropriate sets of genes as well as inappropriate gene activation is typically deleterious to the biological program. Chromatin accessibility is one of the strong indicators for selective transcription factor binding (10,11). However, pioneer factors are capable of binding to inaccessible chromatin (1). Therefore, other chromatin contexts must be involved in selective enhancer formation. Cooperative binding by multiple TFs, partial motif recognition, nucleosome positioning, and chromatin remodeling factors are thought to be involved in this process (3,12–14). BRG1 or BAF complex has been shown to be involved in the establishment of de novo open chromatin regions in various cell contexts (15–20). However, the mechanisms underlying the specific gene targeting and regulation in different genomic contexts are still not fully understood.

CHD4 (Chromodomain Helicase DNA Binding Protein 4) is a catalytic core component of the NuRD (Nucleosome Remodeling and Deacetylase) chromatin remodeling complex and is known to regulate gene expression and DNA damage responses (21,22). CHD4 is involved in multiple developmental processes including neural development and cardiac development (23–25). Similar to other chromatin remodeling factors, genetic and epigenetic data in cancer patients detected frequent alterations in the CHD4 gene suggesting key roles of CHD4 during tumorigenesis and tumor progression (21,26–29). Particularly in breast cancer, chromosome amplification and mRNA up-regulation of CHD4 were observed, and higher expression of CHD4 is associated with poorer patient outcomes in triple negative (or basal) breast cancer cells (30,31). CHD4 knockdown has been shown to inhibit MDA-MB-231 basal breast cancer cell growth in mouse xenograft model (31). Because the NuRD complex contains histone deacetylases such as HDAC1 or HDAC2, it has been thought to act as a gene silencer (32–35). However, the genomic distribution of CHD4 is predominantly enriched at promoters or open chromatin regions, and the CHD4 function appears to be cell context specific (36–39). It is also largely unknown how this gene silencing remodeling complex contributes to cellular reprogramming mediated by pioneer factors (3).

In this study, we measured the impacts of CHD4 depletion in MDA-MB-231 basal breast cancer cells. Characterization of chromatin features by multi-omics methodologies revealed differential impacts of CHD4 knockdown in steady-state cells vs cellular reprogramming processes. CHD4 knockdown in steady-state MDA-MB-231 cells tended to increase chromatin accessibility at promoter-distal regions. Redistribution of AP1 family transcription factors was observed in the absence of CHD4. During the GATA3-induced mesenchymal-to-epithelial transition (MET) cell reprogramming processes (40), CHD4 maintains chromatin architecture mainly at intergenic regions. In the absence of CHD4, chromatin accessibility was increased at closed chromatin, especially at GATA3 binding peaks. Abnormal chromatin opening led to increased expression of genes unrelated to MET. High-resolution nucleosome mapping suggested that CHD4 prevents MET-unrelated gene expression by mediating nucleosome formation over the GATA3 binding sites. These results demonstrate that CHD4, and by extension NuRD complex, monitors transcription factor/chromatin interactions and modulates gene regulation by transcription factors.

## Materials and methods

### Cell line and cell culture

MDA-MB-231 and T47D cells were originally obtained from ATCC. Both cells were cultured in DMEM high-glucose medium with 10% FBS (Thermo Fisher Scientific or R&D Systems). Doxycycline-inducible GATA3 expression system in MDA-MB-231 cells was developed by lentiviral transduction. Ty1-tagged GATA3 gene was inserted into the pINDUCER20 vector, and the lentivirus was generated by the second generation lentiviral plasmids system using 293T cells. pINDUCER20 was a gift from Guang Hu (NIEHS/NIH). psPAX2 and pMD2.G were gifts from Didier Trono (Addgene plasmid #12260, #12259). After antibiotic selection with G418, cell colonies were collected, and GATA3 expression levels were investigated by western blot. The cell clone that has low basal GATA3 expression (without DOX treatment) was used in this study.

The pGIPZ vectors and lentiviruses encoding CHD4 shRNA and control shRNA were provided by the NIEHS Epigenomics Core and Viral Vector Core. 400 000 cells were plated on 6 cm dishes, and infected with these shRNA lentiviruses at 24 and 32 h after plating. After overnight incubation, the medium was replaced with 4 ml of fresh medium and further incubated for 2 days. The GATA3 expression was initiated by adding DOX (1 μg/ml at final) in fresh medium. Twelve hours after induction, cells were harvested and resuspended in PBS.

### ChIP-seq

ChIP-seq libraries were prepared as previously described (41). Briefly, DOX treated cells were fixed at 12 h with 1% formaldehyde. Fixed cells were treated with hypotonic buffer containing 10 mM HEPES-NaOH pH 7.9, 10 mM KCl, 1.5 mM MgCl2, 340 mM sucrose, 10% glycerol, 0.5% Triton X-100 and protease inhibitor cocktail (Thermo Fisher Scientific). Chromatin was digested by sonication with Covaris S220 in the lysis buffer containing 20 mM Tris–HCl pH 8.0, 2 mM EDTA, 0.5 mM EGTA, 0.5 mM PMSF, 5mM sodium butyrate, 0.1% SDS and protease inhibitor cocktail. 2.5 μg of each antibody was added to each chromatin solution (1 million cells/reaction). After overnight incubation, protein A/G mixed Dynabeads were added, and the samples were rotated for 2 h. Eluted DNAs were reverse crosslinked at 65°C for 4 h, followed by the incubation with proteinase K for 1 hour and purified by AMPure XP (Beckman Coulter). For the CHD4 ChIP case, 4 units of Micrococcal nuclease (MNase) were added to the cell lysates, followed by a 3-min incubation at 37°C before sonication.

ChIP-seq libraries were generated by the NEXTflex Rapid DNA-seq kit (PerkinElmer) and sequenced on NextSeq 500 (Illumina, paired-end) at the NIEHS Epigenomics Core Facility. The same data processing protocol to ATAC-seq was used. Mapped reads were converted to a single fragment and used to generate genome coverage tracks on the UCSC Genome Browser and for metaplot analyses. GATA3 ChIP-seq peaks were defined by HOMER v4.1 with default parameters (42). CHD4 ChIP-seq peaks were defined by PeaKDEck (43) using the following parameters: -sig 0.0001 -bin 300 -back 3000 -npBack 2500000. To carry out correlation and differential analyses, we used read counts from the reference peak sets and processed the data using the SARTools pipeline (44) with edgeR (45).

### ATAC-seq

The ATAC-seq libraries were prepared as previously described (41,46). 25 000 cells (in 25 ul) were transferred to new tubes, and nuclei were isolated with CSK buffer (10 mM PIPES pH 6.8, 100 mM NaCl, 300 mM sucrose, 3 mM MgCl_2_, 0.1% Triton X-100). Nuclei were treated with 2.5 μl of Tn5 Transposase (Illumina) in the standard tagmentation reaction buffer (25 μl). A total of 8 PCR cycles were performed to amplify the DNA fragments, and the libraries were sequenced on NextSeq 500 at the NIEHS Epigenomics Core Facility. The raw sequence reads were filtered based on the mean base quality score >20. Adapter sequences were removed by Trim Galorre! (Babraham Institute). Processed reads were mapped to hg19 genome using Bowtie 0.12.8 (47), and uniquely mapped reads (non-duplicate reads) were used for the subsequent analysis.

Peak classification was conducted based on the following criteria. Newly accessible peaks are defined as the GATA3 peaks that show (i) >2-fold increase in ATAC-seq signals at GATA3 peaks (±200 bp from peak center) compared to the control (time 0 h) condition and (ii) >30 normalized reads at the peak flanking (±1 kb) region.

Constitutively accessible peaks are defined as the GATA3 peaks that show >30 normalized reads at the flanking regions (±1 kb). Constitutively inaccessible peaks are defined as the GATA3 peaks that show <30 normalized reads at the flanking regions.

### Capture MNase-seq

Capture MNase-seq was performed as previously described (48). Biotinylated RNA probes were designed and purchased via SureSelect Custom DNA Target Enrichment Probes system with the following probe design specification (tiling density 2×, masking: least stringent, boosting: balanced). The target regions are listed on Supplementary Table S1. Nucleosomal fragments were prepared by digesting nuclei with Micrococcal nuclease (MNase). Sequencing libraries were prepared by NEXTflex Rapid DNA-Seq kit (PerkinElmer). Libraries from different conditions were pooled, and 750 ng DNAs were used to perform nucleosomal DNA fragment enrichment at a subset of GATA3 peaks with the SureSelect Enrichment kit. After the fragment enrichment by RNA probe hybridization and streptavidin pull-down, captured DNAs were amplified by PCR (12 cycles) and sequenced on NextSeq 500 at the NIEHS Epigenomics Core Facility.

The same data processing protocol to ATAC-seq was used for the capture MNase-seq data but duplicate reads were retained. To generate heatmaps, midpoints only from mono-nucleosomal fragments (120–170 bp) were collected. The midpoint frequency (considered as dyad frequency) was normalized by the data from Time 0.

### RNA-seq

Total RNAs were purified by the Qiagen RNeasy kit. Sequencing libraries were generated by TruSeq RNA library preparation kit with Ribo-Zero and sequenced on NextSeq 500 (Illumina) at the NIEHS Epigenomics Core Facility. Filtered read pairs were mapped to hg19 by STAR (version 2.5) with the following parameters: – out-SAMattrIHstart 0 –outFilterType BySJout –alignSJoverhangMin 8 – out-MultimapperOrder Random (49). Subread featureCounts (version 1.5.0–0-p1) and DESeq2 (v1.10.1) with FDR < 0.01 were used to define differentially expressed genes (DEGs) (50). Pathway analysis was performed by DAVID database (51,52) using DEGs that have |log_2_ (fold change)| >0.5. DEGs are summarized in Supplementary Table S2.

### Migration assay

The control shRNA or CHD4 shRNA expressing MDA-MB-231 cells were prepared as described above using the lentiviruses. The GATA3-induced cells were cultured for at least 2 days. Four million were seeded in each 6-well plate and grown overnight. The confluent cells were scratched with a 1 ml micropipette tip. After scratching, the cells were washed with 2 ml of pre-warmed serum-free medium, and the same volume of the serum-free medium was added to each well. Cell images were taken at multiple time points (0, 8, 16, 24 and 33 h) by Olympus IX71 microscope, and imageJ (Version: 2.1.0/1.53c) was used to quantify wound areas.

### Antibodies

Anti-Ty1 antibody (MilliporeSigma, Imprint monoclonal BB2), JUNB (Cell Signaling, C37F9), ATF3 (Cell Signaling, E9J4N), FRA1 (Cell Signaling, D80B4), and CHD4 Abcam (Abcam, ab72418) were used for ChIP-seq and western blot. GATA3 antibody was generated in rabbits using recombinant 6x histidine tag-fused GATA3 full-length wild-type protein (53).

### Statistical analysis

**Figure 1.**
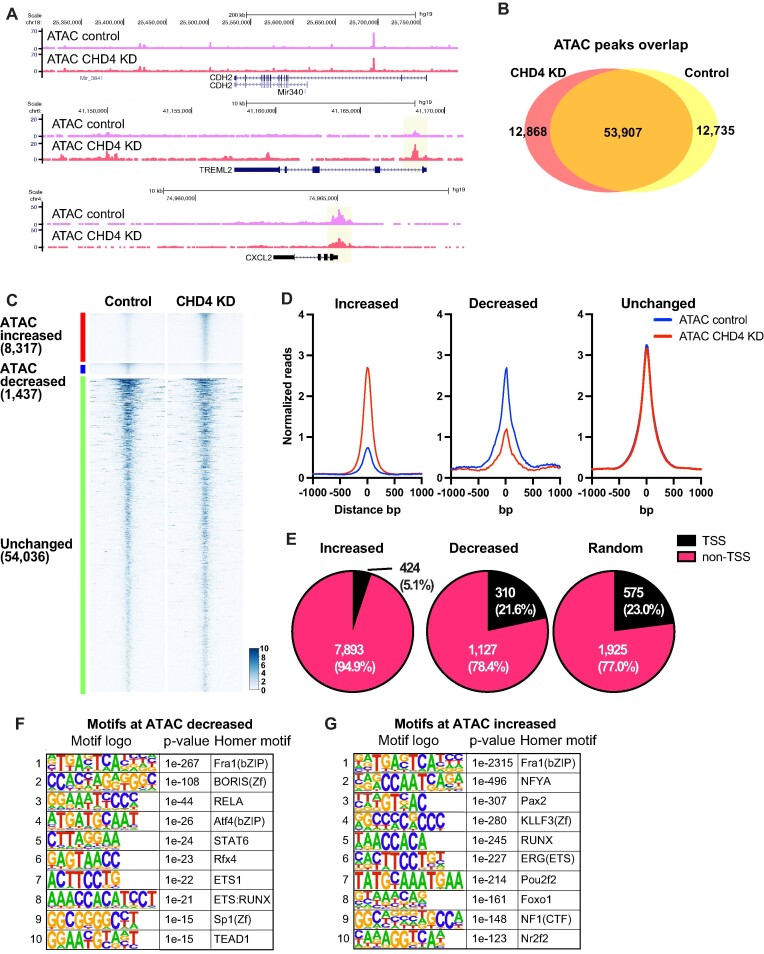
CHD4 modulates chromatin accessibility at promoters in MDA-MB-231. (**A**) Genome browser tracks showing ATAC-seq signals in control or CHD4 knockdown (KD) cells. Unchanged, increased, and decreased regions are selected. (**B**) Venn diagram showing the ATAC-seq peak overlap between control or CHD4 KD cells. (**C**) edgeR differential ATAC-seq peak analysis in CHD4 KD cells. FDR <0.01 and |fold change| >2 are applied to define differential peaks. Heatmap showing ATAC-seq signals at increased, decreased, or unchanged ATAC-seq peaks. (**D**) Metaplot showing normalized ATAC-seq reads/peak at differential peaks. (**E**) Pie chart showing the frequency of TSS and non-TSS peaks in each peak group. (F, G) HOMER de novo motif analysis. Decreased (**F**) or increased ATAC-seq peaks (**G**) are used as input.

**Figure 2.**
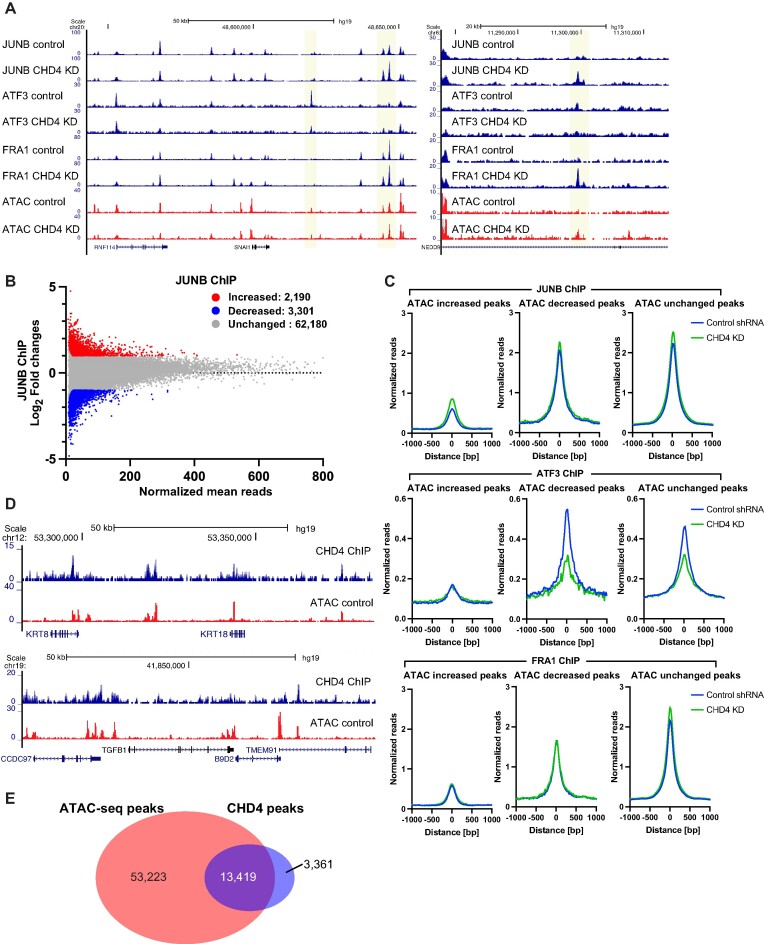
AP1 family transcription factors are redistributed following CHD4 depletion. (**A**) Genome browser tracks showing JUNB, ATF3, and FRA1 ChIP-seq data. Differential peaks between control and CHD4 KD data are highlighted in yellow. ATAC-seq data are also shown as a reference for open chromatin regions. (**B**) Scatter plot showing increased (red), decreased (blue), and unchanged (grey) JUNB ChIP-seq peaks. FDR < 0.01 and |fold change| > 2 are applied to define differential peaks by edgeR. (**C**) Metaplot showing normalized ChIP-seq reads/peak at ATAC-seq differential peaks. JUNB (top). ATF3 (middle), and FRA1 (bottom) ChIP-seq signals in control (blue) or CHD4 KD cells (green) are plotted. (**D**) Genome browser tracks showing the frequent overlap between CHD4 and ATAC-seq peaks. CHD4 ChIP-seq were performed in control MDA-MB-231 cells. (**E**) Venn diagram showing the overlap between ATAC-seq peaks and CHD4 peaks.

To calculate adjusted *P*-values in differential peak and gene expression analysis, the Benjamini–Hochberg method was used (Figures 1C, 2B, 4A, 5C and D, Supplementary Figure S2A, B). For the cell migration assay shown in Figure 6D, the *t*-test was used to calculate *P*-values. The Mann–Whitney test was used for box plot comparison (Supplementary Figure S2E–G).

## Results

### CHD4 depletion mediates altered chromatin accessibility and gene expression

To explore the range of chromatin features regulated by CHD4 in somatic cells, we depleted it in MDA-MB-231 basal breast cancer cells using shRNA (Supplementary Figure S1A) followed by genome-wide analysis of chromatin accessibility using ATAC-seq (46,54). CHD4 has been shown to regulate tumor growth in MDA-MB-231 cells (31). The physical location of most ATAC-seq peaks in the genome was unchanged, while approximately 1/3 of detected peaks were either lost or gained following CHD4 depletion (Figure 1A, B). Comparisons at the level of individual biological replicates indicated that loss of CHD4 rather than inherent variability drives the outcome of this comparison (Supplementary Figure S1B, C).

In addition to loss or gain of individual peaks, we performed edgeR differential peak analysis to assess whether individual loci had changes in the level of accessibility with or without a change in location. Somewhat paradoxically, most altered ATAC peaks exhibit an increase in transposition following depletion of CHD4 (Figure 1C, D). We further confirmed that these changes are specific to CHD4 depletion rather than technical or biological variations (Supplementary Figure S1D–F). The loci with increased accessibility were largely confined to peaks located greater than 1 kb from an annotated transcription start site (TSS), and this distribution differed from random (*P*-value = 0.00001, chi square test) (Figure 1E, Supplementary Figure S1G). Peaks with decreased accessibility were associated with transcription start sites at a frequency similar to a random peak set (Figure 1E). These results suggest that CHD4 may have a different impact on chromatin architecture at promoters than at distal regulatory elements.

We asked whether the alteration in peak intensity reflected changes in the binding behavior of individual transcription factors by assessing the enrichment of these loci for known transcription factor binding motifs using HOMER (42). Surprisingly, there was considerable overlap in the binding motifs enriched in the peaks with increased accessibility and the motifs enriched in the peaks with decreased accessibility. Motifs for the AP1 family, the ETS family and the RUNX family were present in both enriched motif sets, with an AP1 motif being the most enriched in both sets (Figure 1F, G). To further access AP1 motif enrichment, we performed the HOMER motif enrichment analysis using the gained (12 868 ATAC-seq peaks, uniquely observed in CHD4 KD cells) and lost peaks (12 735 ATAC-seq peaks, only observed in control cells) defined by peak overlap analysis shown in Figure 1B. Similarly, motifs for the AP1 family members were significantly enriched at both gained and lost peaks (Supplementary Figure S1H, I). This outcome was unexpected, as it suggested that loss of CHD4 leads to change in binding site selection by transcription factors.

To assess whether individual transcription factors were, in fact, relocalized in the genome, we performed ChIP-seq for AP1 family members, JUNB, FRA1 and ATF3 with and without depletion of CHD4. All tested AP1 family members showed thousands of differential bindings upon CHD4 depletion (Figure 2A). For the case of JUNB, 2190 increased and 3301 decreased peaks were observed (Figure 2B, C). Slightly smaller numbers of differential binding peaks were observed for FRA1 and ATF3 (Supplementary Figure S2A, B). Metaplot analysis at ATAC-seq differential peaks suggested differential impacts of CHD4 depletion on JUNB, FRA1 and ATF3 binding (Figure 2C, Supplementary Figure S2C). JUNB binding was higher in CHD4 KD cells at both increased and unchanged ATAC-seq peaks, whereas ATF3 binding was lower in these cells at both decreased and unchanged peaks. Subtle changes were observed in FRA1 ChIP-seq data. These results suggest that although AP1 family members may not be primary factors in modulating chromatin accessibility upon CHD4 depletion, CHD4 still regulates the chromatin binding activities of AP1 family transcription factors.

To ask whether these alterations in chromatin were associated with CHD4 binding, we performed ChIP-seq for CHD4 in MDA-MB-231 cells. While CHD4 ChIP-seq showed reduced signal-to-noise ratios compared to ATAC-seq, the data had sufficient quality for 16 780 peaks to be detected by the PeaKDEck peak calling (43). CHD4 peaks were frequently observed at open chromatin regions (Figure 2D), and approximately 80% of CHD4 peaks (13 419 peaks out of 16 780 peaks) overlapped with ATAC-seq peaks in MDA-MB-231 cells (Figure 2E). The frequency of overlap between CHD4 and ATAC-seq peaks, as well as the ATAC-seq signals at CHD4 peaks, were significantly higher than those in randomly selected genomic regions (Supplementary Figure S2E, F).

**Figure 3.**
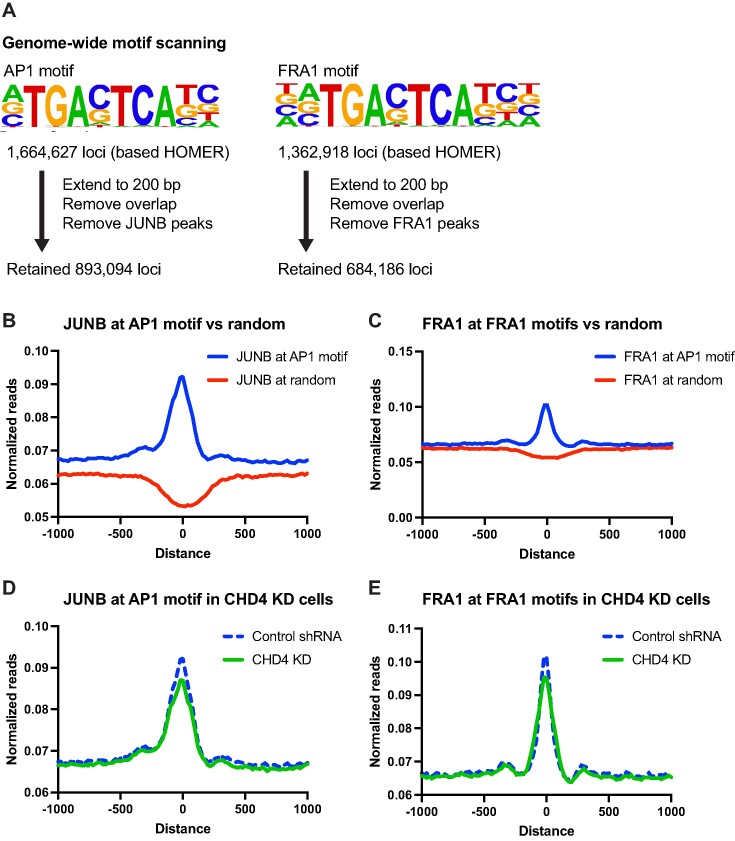
Genome-wide motif sampling of AP1 family proteins is affected by CHD4 knockdown. (**A**) Scheme for defining potential binding target loci. HOMER AP1 or FRA1 motif-containing sites are obtained from the HOMER database. For the downstream analysis, each consensus motif locus was extended to 200 bp, and the overlaps within the consensus motif loci and with the observed JUNB or FRA1 ChIP-seq peaks were removed. The retained loci were used for detecting motif sampling activities of JUNB and FRA1. (**B**) Metaplot showing JUNB ChIP-seq signals at AP1 consensus motif (blue) or randomly selected genomic (red) regions. (**C**) Metaplot showing FRA1 ChIP-seq signals at FRA1 consensus motif (blue) or randomly selected genomic (red) regions. (D, E) Metaplot showing JUNB (**D**) or FRA1 (**E**) ChIP-seq signals at consensus motif regions in control (blue) or CHD4 KD (green) cells.

Differential chromatin accessibility and redistribution of multiple AP1 family members upon CHD4 depletion suggested that CHD4 is important for target site selection by transcription factors. AP-1 proteins have a high affinity to the palindromic sequence 5′-TGA G/C TCA-3′, but transcription factors are known to possess nonspecific or non-consensus DNA binding. Since AP1 motifs are enriched at both increased and decreased ATAC-seq peaks, it is possible that CHD4 regulates the sequence-specific binding activities (motif sampling) of transcription factors. Our peak-centered analyses in Figure 2 may have excluded weaker binding events. To account for all potential binding events, including those with weaker binding, we performed metaplot analyses of JUNB and FRA1 at all potential binding sites identified based on sequence match (Figure 3A). In the HOMER database, we found >1 million AP-1 potential binding sites. We first tested if we could detect consensus motif binding of JUNB and FRA1 outside of ChIP-seq peaks. We first excluded the observed ChIP-seq peaks to minimize the bias in the ChIP-seq data resulting from motif-independent binding or indirect binding caused by cell fixation (Figure 3A). Both JUNB and FRA1 exhibited significantly stronger enrichment at loci containing the consensus motif compared to randomly selected genomic regions (Figure 3B, C, Supplementary Figure S2F), suggesting that this analysis allows us to capture motif sampling by JUNB and FRA1. When CHD4 was depleted, JUNB and FRA1 binding signals were significantly decreased at the consensus motif sites (Figure 3D, E, Supplementary Figure S2G). These results suggest that CHD4 potentially modulates motif sampling activities of JUNB and FRA1, potentially permitting a more promiscuous sampling process.

**Figure 4.**
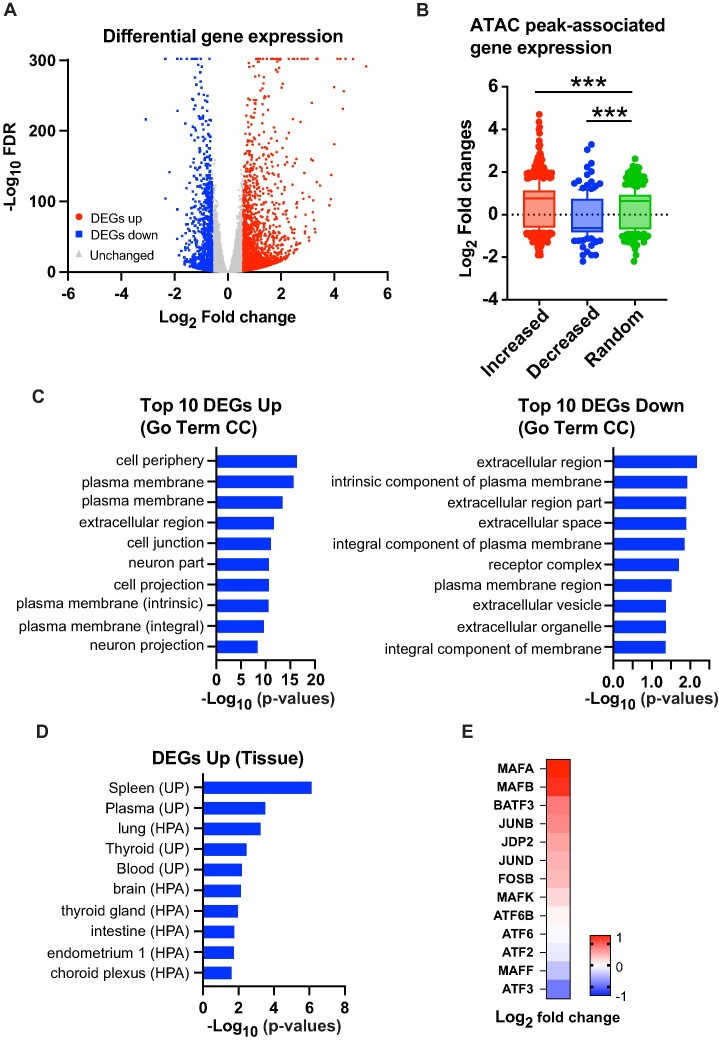
CHD4 knockdown results in aberrant gene expression unrelated to breast cancer program. (**A**) Volcano plot showing differential gene expression upon CHD4 depletion. FDR < 0.05 and |fold change| > 1.5 were applied to define differentially expressed genes. (**B**) Gene expression of ATAC-seq differential peak associated genes. Box plots show log_2_ fold changes from each peak group. Increased, decreased, and randomly selected unchanged peaks are assigned to closest genes. (**C**) Pathway enrichment analysis. Most significantly altered genes (fold change ± 2) were used for Go term Cellular Component (CC) analysis by DAVID (51,52). Top 10 pathways are indicated. (**D**) Functional annotation of up-regulated genes in CHD4 KD cells. DAVID tissue expression annotation was performed using up-regulated genes. (**E**) Gene expression of AP1 family proteins. ATAC-seq differential peak associated genes. Heatmap shows log_2_ fold changes in CHD4 KD cells compared to control shRNA condition.

To examine the functional consequences to gene expression of CHD4 depletion, we performed RNA-seq. FDR < 0.05 and fold change criteria (|fold change| > 1.5) were used to identify significantly altered transcripts. 1880 genes were upregulated and 1071 genes were downregulated in steady state MDA-MB-231 cells (Figure 4A). The number of genes altered in steady state transcript level was also skewed towards gene activation, but not to the extent as alterations in transposition. Integration of ATAC data and gene expression indicated that genes associated with increased transposition had higher steady state transcript levels following CHD4 depletion while genes with decreased accessibility had lower levels of transcript (Figure 4B). To further assess whether the observed changes are associated with CHD4 chromatin binding, we analyzed the overlap between differentially expressed genes (DEGs) and the peaks identified in CHD4 ChIP-seq data. For this analysis, we selected the gene closest to each CHD4 peak within a 100 kbp range (Supplementary Table S3). Of the 1880 up-regulated genes identified, 506 overlapped with CHD4-associated genes. Similarly, among the 1071 down-regulated genes, 302 were found to be associated with CHD4. These findings suggest a potential direct regulatory influence of CHD4 on approximately 20–30% of the DEGs. Gene ontology analysis suggested that altered transcript levels were enriched at genes involved in plasma membrane processes and interaction with the extracellular space (Figure 4C). Somewhat surprisingly, upregulated transcripts were associated with tissues other than breast (Figure 4D), suggesting that loss of CHD4 leads to loss of cell-type specificity in the transcriptional program. Direct inspection of the RNA-seq data revealed that the expression levels of multiple transcription factors were altered upon CHD4 depletion including AP1 family members (Figure 4E). Therefore, the observed changes in AP1 family member expression could contribute to the differential chromatin binding of those proteins shown in Figures 2 and 3.

### CHD4 antagonizes enhancer formation by GATA3

We observed alterations in chromatin accessibility and transcription factors’ binding upon depletion of CHD4. However, we also observed changes in the expression of transcription factor family members, potentially complicating the analysis and data interpretation. Therefore, we moved to a more defined system with a temporal component, transcription-factor dependent cellular reprogramming. We established a doxycycline-inducible GATA3 expression system in MDA-MB-231 mesenchymal breast cancer cells. GATA3 expression in MDA-MB-231 cells has been shown to induce mesenchymal to epithelial transition (MET) (40,55). We selected a cell clone that shows minimal GATA3 expression in the absence of doxycycline (hereafter DOX) but expresses biologically relevant amounts of GATA3 protein upon DOX treatment. In this cell clone, GATA3 protein expression was observed within 3 h after the addition of DOX to the media and was saturated by 12 h (Supplementary Figure S3A). Stable GATA3 expression was observed for at least 48 h after DOX induction.

**Figure 5.**
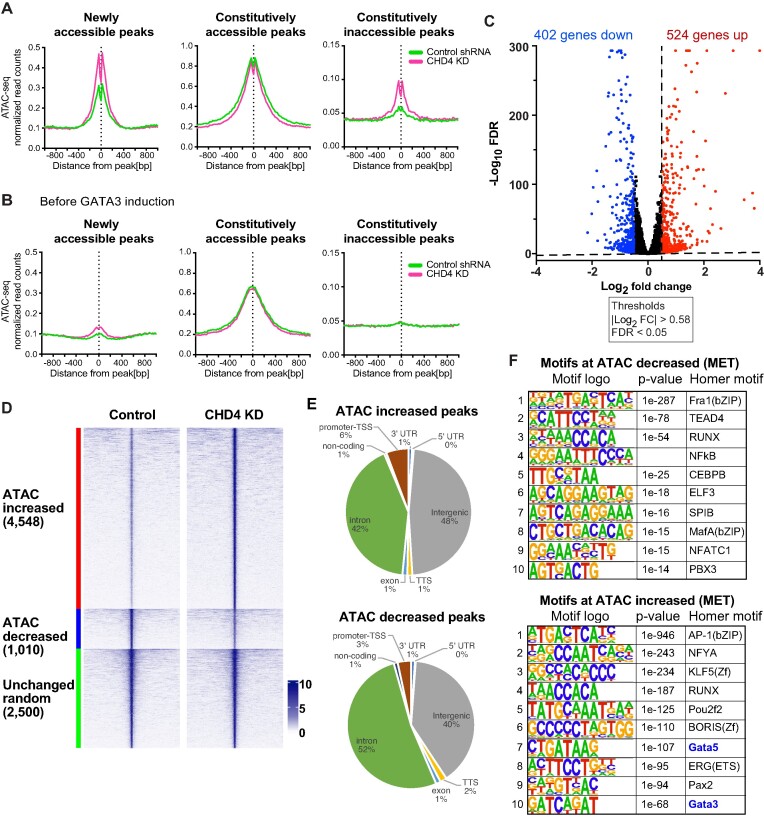
CHD4 knockdown leads to abnormal chromatin opening. (**A, B**) Metaplots showing ATAC-seq signals in the control shRNA or CHD4 shRNA transduced cells. Averaged ATAC-seq signals before (b) or after (a) GATA3 expression are plotted in each peak group. (**C**) Volcano plot showing differential gene expression 12 h after GATA3 expression. Up- and down-regulated genes (FDR < 0.05, |log_2_ (fold change)| > 0.5) are highlighted in red and blue, respectively. (**D**) ATAC-seq differential peak analysis upon CHD4 knockdown in MET. RNAs were collected 12 h after GATA3 expression. FDR < 0.01 and |fold change| > 2 are applied to define differential peaks. Heatmap shows ATAC-seq signal intensity in control or CHD4 KD cells at increased, decreased, and randomly selected unchanged peaks. ATAC-seq signals at increased, decreased or randomly selected unchanged ATAC-seq peaks. (**E**) Pie chart showing peak annotation defined by HOMER. Increased or decreased ATAC-seq peaks are classified into 8 peak categories. (**F**) HOMER de novo motif analysis. Decreased (top) or increased (bottom) ATAC-seq peaks upon CHD4 KD in MET condition are used as input.

We collected ATAC-seq, CHD4 and GATA3 ChIP-seq and RNA-seq 12 h after DOX treatment to characterize GATA3 and CHD4-dependent changes in chromatin architecture and gene expression. By comparing transposase accessibility at GATA3 peaks before and after GATA3 induction, we characterized three predominant types of loci: loci that transition from inaccessible to accessible (newly accessible), loci where GATA3 binding occurs within transposase accessible chromatin (constitutively accessible) and loci where GATA3 binds to inaccessible chromatin that remains inaccessible (constitutively inaccessible) following GATA3 expression (Figure 5A, B, green curves). CHD4 was clearly recruited at GATA3 binding sites (Supplementary Figure S3B, C). While we observed an increase in CHD4 binding across all three peak groups, the overlap between CHD4 and GATA3 at newly accessible and constitutively accessible GATA3 peaks was substantial (Supplementary Figure S3B). RNA-seq analysis revealed that 926 genes were altered in steady state abundance following GATA3 expression, with slightly more genes being activated (524) than decreased (402) (Figure 5C). Consistent with previous reports (40), transcripts with altered levels were enriched in categories involved in mesenchymal to epithelial transition (Supplementary Figure S3D). To further confirm the overlap between GATA3 and CHD4 in breast cancer cells, we performed CHD4 ChIP-seq in T47D cells, where both CHD4 and GATA3 are endogenously expressed (Supplementary Figure S3E). Among the 34 380 GATA3 peaks that we had previously defined (41,56), CHD4 signals were observed at 16 225 GATA3 peaks (47%), confirming the frequent overlap between CHD4 and GATA3 in luminal breast cancer cells.

When CHD4 was depleted in this system, we observed striking alterations in several features. Of loci exhibiting significant alterations in ATAC sensitivity, 82% (4548 of 5558) demonstrated an increase in accessibility (Figure 5D), similar to control MB-MDA-231 cells (Figure 1C). Unlike the case in control cells where increased accessibility was overwhelmingly at distal elements and decreased accessibility was somewhat more balanced, changes in transposition in the reprogramming system were overwhelmingly found distant from annotated transcription start sites (Figure 5E). Globally, altered ATAC loci were enriched in AP1 and RUNX motifs, with GATA motifs enriched at loci that gain accessibility (Figure 5F). When we focused analysis on loci with GATA3 peaks, we found that depletion of CHD4 led to a substantial increase in accessibility at newly accessible peaks where GATA3 binding leads to enhancer licensing (Figure 5A) (40). Surprisingly, peaks where GATA3 fails to induce chromatin opening in the presence of CHD4 frequently display increased accessibility in its absence (Figure 5A), suggesting that CHD4 acts to oppose the chromatin-opening ability of GATA3.

**Figure 6.**
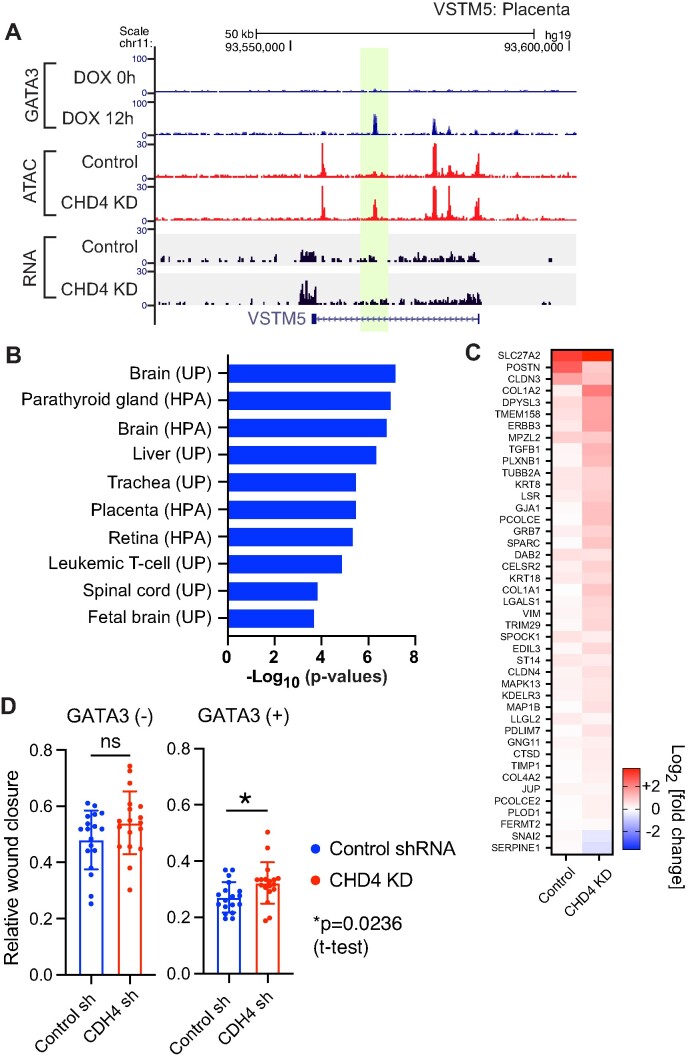
CHD4 regulates enhancer activities during MET cell reprogramming. (**A**) An example of aberrant gene expression at a constitutively inaccessible GATA3 peak. Genome browser tracks show an example of up-regulated genes associated with the constitutively inaccessible GATA3 peaks. ATAC-seq and RNA-seq was performed 12 h after GATA3 induction. The *de novo* open chromatin site upon CHD4 KD is highlighted in yellow. VSTM5 is known to be expressed in brain and placenta. (**B**) Functional annotation of the constitutively inaccessible peak associated genes. DAVID tissue expression annotation was performed using the up-regulated genes that are associated (±100 kb) with the constitutively inaccessible GATA3 peaks. (**C**) Heatmap showing expression levels of MET associated genes. Fold changes were calculated based on the DESeq2 gene counts in the GATA3-expressed (12 h after DOX treatment) control or CHD4 knockdown cells compared to DOX minus condition. (**D**) Bar graphs showing the relative wound closure in the wound healing assay. The wound healing assay was performed in the control or CHD4 knockdown cells before and after GATA3 expression (16 h). The average values are shown with SDs (*N* = 18, 3 biological replicates x 6 technical replicates).

RNA-seq performed following CHD4 knockdown revealed MET-unrelated gene expression at loci linked to constitutively inaccessible sites, exemplar genes are depicted in Figure 6A and Supplementary Figure S4. At such loci, GATA3 binding is not associated with transposase accessibility in the presence of CHD4, in its absence accessibility is evident along with increased transcript level. Gene ontology analysis revealed that the up-regulated genes associated with the constitutively inaccessible peaks were significantly enriched with tissue-specific genes related to GATA3 function in cell contexts other than breast (Figure 6B). For instance, GATA3 is known to be important for trophoblast differentiation and placental development (57,58). Placenta related genes such as VSTM5, NDNF and HAPLN1 were up-regulated in the CHD4 knockdown cells (Figure 5A, Supplementary Figure S4).

CHD4 depletion also impacted the biological outcome of GATA3-mediated cell reprogramming, mesenchymal to epithelial transition. MET-related gene expression was exacerbated in the CHD4 knockdown cells (Figure 6C), which is consistent with the increased ATAC-seq signals at newly accessible peaks after CHD4 knockdown. Cell migration assays indicate that GATA3 expression in the CHD4 knockdown cells still showed MET phenotypes at the cellular level, but the degree of cell migration was modestly impacted by CHD4 knockdown (Figure 6D). These results suggest that CHD4 acts to constrain the ability of GATA3 to bind its motif and elicit alterations in gene expression and cellular phenotype.

### CHD4 promotes nucleosome formation over transcription factor motifs

**Figure 7.**
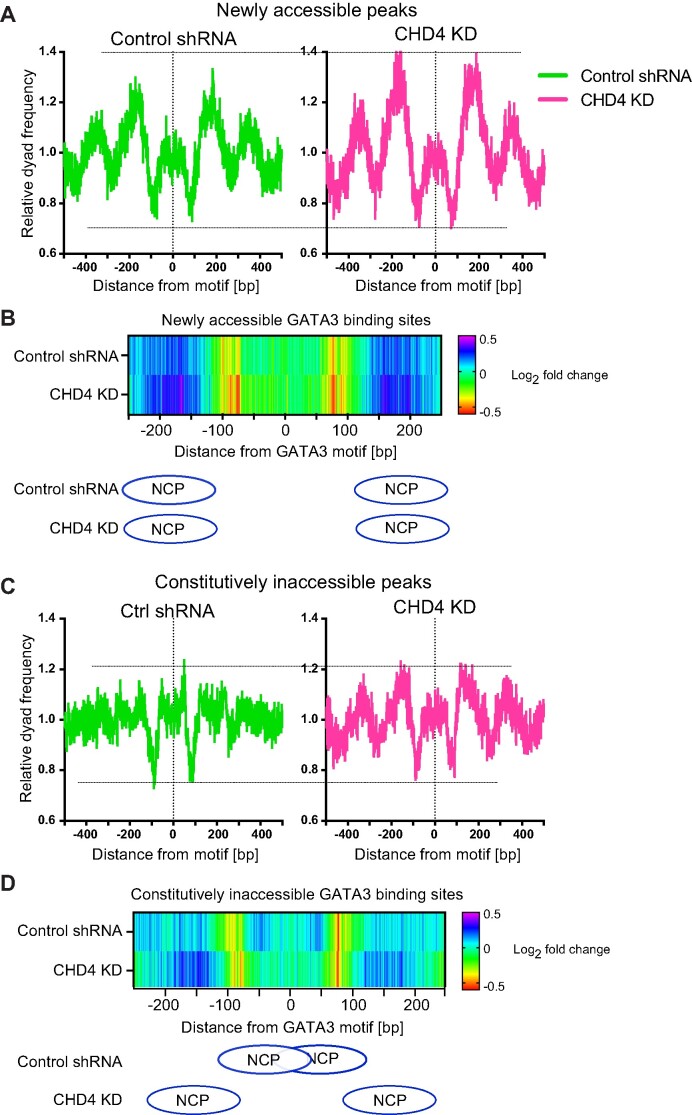
High-resolution nucleosome mapping reveals aberrant nucleosome remodeling induced by CHD4 knockdown. (**A**) Metaplot showing averaged dyad frequency at newly accessible peaks in control or CHD4 knockdown cells. The conventional MNase-seq was performed in the control or CHD4 knockdown cells. (**B**) Capture MNase-seq results at the 750 selected newly accessible peaks. Heatmap shows dyad frequency at newly accessible sites relative to time 0 h. Ellipses indicate the most enriched nucleosome positioning at the GATA3 motif flanking region. (**C**) Metaplot showing averaged dyad frequency at constitutively inaccessible peaks in control or CHD4 knockdown cells. The nucleosome fragments were collected by the conventional MNase-seq. (**D**) Capture MNase-seq data at the 750 selected constitutively inaccessible peaks. Heatmap shows dyad frequency at constitutively inaccessible sites relative to time 0 h. Ellipses indicate the most enriched nucleosome positions in control (top) or CHD4 knockdown (bottom) cells.

We previously reported that nucleosome remodeling patterns are associated with the chromatin opening activities of GATA3 (40,48). At loci that become accessible following GATA3 induction (newly accessible sites), nucleosome depletion was observed at the center of GATA3 binding peaks, while nucleosome repositioning and accumulation were observed at the constitutively inaccessible GATA3 binding peaks. To understand the impact of CHD4 depletion on nucleosome remodeling following induction of GATA3, we performed MNase-seq to map nucleosomes during the GATA3-mediated cellular reprogramming. Newly accessible peaks exhibit the characteristic pattern of MNase-resistant, positioned nucleosomes flanking a moderately resistant region centered over the transcription factor binding motif (Figure 7A, left panel). When we deplete CHD4, the pattern observed remains unchanged while the amplitude of nucleosome peaks flanking the GATA3 binding site increases. This result suggests that a larger number of alleles within the population sampled have productively bound GATA3, creating a phased array of nucleosomes. To gain further insight into GATA3-induced nucleosome remodeling, we turned to a higher resolution technique, capture MNase-seq, which provides deep mapping of nucleosome positions at individual GATA3 binding loci (Supplementary Figure S5A). In this technique, custom designed biotinylated RNA probes are used to enrich defined loci. We designed the RNA probes against ∼2700 genomic intervals, which contain ∼1800 GATA3 binding sites and ∼940 negative control loci where GATA3 fails to accumulate. Mono-nucleosomal fragment midpoint frequency (dyad frequency) was calculated to monitor nucleosome remodeling during GATA3-mediated reprogramming. In the absence of CHD4, we observed a substantial increase in nucleosomal dyads flanking the GATA motif with no alteration in the final position of the nucleosomes (Figure 7B, Supplementary Figure S5B). These results were consistent with the data from the conventional MNase-seq and suggest that in the absence of CHD4, more alleles within the population sampled exhibit productive binding of GATA3 and create a phased nucleosomal array flanking the binding site. This remodeling is not accompanied by a notable increase in the width of the nucleosome-depleted region.

At constitutively inaccessible GATA3 binding sites, we observed a completely different outcome. In conventional MNase-seq, these GATA3-bound loci do not exhibit the phased nucleososmes flanking the binding site that are evident in newly accessible peaks. However, upon CHD4 depletion, we observe a clear pattern of phased nucleosomes flanking the GATA3 binding site that resembles the pattern observed in newly accessible sites (Figure 7C). Capture MNase-seq confirms this observation, showing movement of nucleosomal dyads away from sites where the GATA3 motif is located within the confines of the nucleosome to sites distant from the GATA motif (Figure 7D, Supplementary Figure S5C). At these loci, CHD4 alters the outcome of GATA3 interaction with the chromatin fiber. The data suggested that loss of CHD4 changes the outcome from GATA3 bound to the surface of a nucleosome to GATA3-mediated nucleosome eviction from the binding site and establishment of a phased array of flanking nucleosomes.

## Discussion

TFs face multiple challenges in establishing new gene regulatory networks during development, in response to physiologic or environmental signals, and during *in vitro* cellular reprogramming. These proteins must find appropriate recognition motifs within the genome and they also must contend with physical barriers including chromatin (59–61). In eukaryotes with large genomes, including humans, TFs with short recognition motifs must find the correct loci, and only the correct loci, amongst the potentially millions of matches to their binding motif. In theory, degenerate hexameric binding motifs, such as the WGATAR consensus binding motif for GATA3 (62), should be present about every 500 bp. Based on the human reference genome sequence (hg19), more than 7 million loci contain the GATA3 consensus motif (8). In most cases, TFs are present on the order of thousands to tens of thousands of molecules per nucleus, meaning there are roughly 100-fold more potential binding sites than TFs (5–7). In fact, the number of GATA3 consensus motifs identified in the GATA3 ChIP-seq data from MDA-MB-231 cells (40) is approximately 52 000, which represents <1% of the potential binding motifs.

Chromatin represents a first-line barrier to inappropriate binding of TFs, and it presents a barrier in multiple ways. A subset of TFs cannot read DNA sequences and bind productively to DNA wrapped around a histone octamer (1,60). Within the context of a nucleosome, the rotational and translational phasing of DNA necessarily obscures some chemical information where it closely approximates the histone octamer surface, making it unavailable for sequence readers (12,60). Biochemical and structural data indicate that the location of binding motifs near the center versus near the periphery of a nucleosome has a strong influence on binding and stability (48,60,63,64). Higher order structural features of chromatin, including linker histones, assembly into heterochromatin, or partitioning into low contact frequency nuclear compartments are likely to further restrict the available sequence space to be searched by TFs (61,65). Clearly, other factors must contribute to narrowing the search space and increasing the probability that cellular signals will result in activation (or repression) of the correct subset of genes (19).

CHD4 is important for maintenance of chromatin architecture and is known to suppress gene expression during tissue development, cell differentiation and cell reprogramming (25,32,33,66,67). However, genome-wide mapping finds frequent localization of CHD4 at active gene promoters and enhancers (36,39,68,69), clouding understanding of mechanistic roles by which CHD4 regulates gene expression. In this study, we investigated the roles of CHD4 in steady state basal breast cancer cells and during mesenchymal-to-epithelial transition (MET). In the steady state, CHD4 depletion resulted in a primarily increased chromatin accessibility leading to abnormal gene expression unrelated to breast cancer cell identity. While the loci with decreased chromatin accessibility by CHD4 depletion are enriched at promoters, increased chromatin accessibility regions are enriched at intergenic regions. Motif analysis at differential peaks suggested that CHD4 modulates chromatin binding of multiple AP1 family members. In fact, when CHD4 was depleted, at least three AP1 family proteins, JUNB, ATF3 and FRA1, were redistributed. This finding is consistent with the previous observation in the mouse embryonic stem cells where Mbd3 restoration led to altered chromatin binding by pluripotency-associated transcription factors (70).

During GATA3-mediated MET cell reprogramming, CHD4 knockdown again resulted largely chromatin opening. At both newly accessible and constitutively inaccessible GATA3 bound loci, we observed evidence for alterations in local nucleosome positioning, consistent with active roles for chromatin remodelers. Further investigation revealed that depletion of the chromatin remodeling enzyme CHD4, a core subunit of the NuRD complex, dramatically altered this outcome leading to new accessibility at previously inaccessible loci and increasing accessibility to transposition at accessible loci. This abnormal chromatin opening, not observed in the presence of CHD4, was associated with altered gene expression and affected cell fate transition at the cellular level. We speculate that our observations reflect a general property attributable to CHD4, and by extension to NuRD complex. NuRD is found with high frequency at open chromatin or enhancers (36,39,68,69) where it is integral to the process of enhancer decommissioning during development (23,32,68) and reprogramming (66,67). We propose that NuRD acts, in part, to antagonize transcription factor driven increases in chromatin accessibility – regardless of the ultimate outcome. It seems plausible that local translational motion of nucleosomes relative to transcription factor binding motifs leads to architectural obstacles to motif recognition. At loci that become accessible, co-binding of multiple TFs or recruitment of other chromatin modification/remodeling enzymes generates a competition between factors promoting and opposing DNA accessibility that ultimately reaches a dynamic equilibrium in which some alleles within the population are accessible to structural probes. At loci that fail to become accessible, failure to recruit activating co-factors leads to generation of a very different type of equilibrium, one in which nucleosome translational position relative to the GATA motif permits GATA3 binding to nucleosomal DNA in the absence of detectable accessibility. In this manner, choice of binding sites within the genome can be ‘proof-read’ by chromatin remodelers simply through enforcing a highly dynamic state (71,72). In principle, such chromatin dynamics would promote two critical outcomes: they would provide assurance against inappropriate transcriptional activation following transcription factor binding at incorrect sites and they would provide an opportunity for rapid decommissioning of enhancers to enable progression to new patterns of gene expression consistent with cellular needs. It is still unclear how chromatin remodeling factors can distinguish ‘appropriate’ or ‘inappropriate’ enhancers in steady state cells or during cell reprogramming. A limitation of our study is the potential for indirect effects arising from the loss of CHD4, especially given the complex nature of the CHD4 or NuRD complex, which includes multiple components such as histone deacetylases HDAC1/2. Further studies are necessary to understand the fundamental mechanisms underpinning the action of CHD4 action including its recruitment to specific regulatory regions.

## Supplementary Material

gkae025_Supplemental_Files

## Data Availability

All genomics data have been uploaded to GEO under the following accession numbers: GSE201797, GSE201798, GSE201799, GSE201800, GSE201801. GATA3 ChIP-seq data in the stable cell line (GSE72141) were previously generated (40).

## References

[1] Iwafuchi-Doi M., Zaret K.S. Pioneer transcription factors in cell reprogramming. Genes Dev. 2014; 28:2679–2692. 25512556 10.1101/gad.253443.114 PMC4265672

[2] Cirillo L.A., Lin F.R., Cuesta I., Friedman D., Jarnik M., Zaret K.S. Opening of compacted chromatin by early developmental transcription factors HNF3 (FoxA) and GATA-4. Mol. Cell. 2002; 9:279–289. 11864602 10.1016/s1097-2765(02)00459-8

[3] Swinstead E.E., Paakinaho V., Presman D.M., Hager G.L. Pioneer factors and ATP-dependent chromatin remodeling factors interact dynamically: a new perspective: multiple transcription factors can effect chromatin pioneer functions through dynamic interactions with ATP-dependent chromatin remodeling factors. Bioessays. 2016; 38:1150–1157. 27633730 10.1002/bies.201600137 PMC6319265

[4] Zaret K.S. Pioneer transcription factors initiating gene network changes. Annu. Rev. Genet. 2020; 54:367–385. 32886547 10.1146/annurev-genet-030220-015007 PMC7900943

[5] Biggin M.D. Animal transcription networks as highly connected, quantitative continua. Dev. Cell. 2011; 21:611–626. 22014521 10.1016/j.devcel.2011.09.008

[6] Schwanhäusser B., Busse D., Li N., Dittmar G., Schuchhardt J., Wolf J., Chen W., Selbach M. Global quantification of mammalian gene expression control. Nature. 2011; 473:337–342. 21593866 10.1038/nature10098

[7] Pratt H.E., Andrews G.R., Phalke N., Purcaro M.J., van der Velde A., Moore J.E., Weng Z. Factorbook: an updated catalog of transcription factor motifs and candidate regulatory motif sites. Nucleic Acids Res. 2022; 50:D141–d149. 34755879 10.1093/nar/gkab1039 PMC8728199

[8] ENCODE Project Consortium An integrated encyclopedia of DNA elements in the human genome. Nature. 2012; 489:57–74. 22955616 10.1038/nature11247 PMC3439153

[9] Euskirchen G.M., Rozowsky J.S., Wei C.L., Lee W.H., Zhang Z.D., Hartman S., Emanuelsson O., Stolc V., Weissman S., Gerstein M.B. et al. Mapping of transcription factor binding regions in mammalian cells by ChIP: comparison of array- and sequencing-based technologies. Genome Res. 2007; 17:898–909. 17568005 10.1101/gr.5583007 PMC1891348

[10] Natarajan A., Yardimci G.G., Sheffield N.C., Crawford G.E., Ohler U. Predicting cell-type-specific gene expression from regions of open chromatin. Genome Res. 2012; 22:1711–1722. 22955983 10.1101/gr.135129.111 PMC3431488

[11] Keilwagen J., Posch S., Grau J. Accurate prediction of cell type-specific transcription factor binding. Genome Biol. 2019; 20:9. 30630522 10.1186/s13059-018-1614-y PMC6327544

[12] Michael A.K., Thomä N.H. Reading the chromatinized genome. Cell. 2021; 184:3599–3611. 34146479 10.1016/j.cell.2021.05.029

[13] Mayran A., Drouin J. Pioneer transcription factors shape the epigenetic landscape. J. Biol. Chem. 2018; 293:13795–13804. 29507097 10.1074/jbc.R117.001232 PMC6130937

[14] Iwafuchi-Doi M. The mechanistic basis for chromatin regulation by pioneer transcription factors. Wiley Interdiscipl. Rev. Syst. Biol. Med. 2019; 11:e1427. 10.1002/wsbm.1427 PMC6585746 29949240

[15] King H.W., Klose R.J. The pioneer factor OCT4 requires the chromatin remodeller BRG1 to support gene regulatory element function in mouse embryonic stem cells. eLife. 2017; 6:e22631. 28287392 10.7554/eLife.22631 PMC5400504

[16] Lazar J.E., Stehling-Sun S., Nandakumar V., Wang H., Chee D.R., Howard N.P., Acosta R., Dunn D., Diegel M., Neri F. et al. Global regulatory DNA potentiation by SMARCA4 propagates to selective gene expression programs via domain-level remodeling. Cell Rep. 2020; 31:107676. 32460018 10.1016/j.celrep.2020.107676

[17] Xiao L., Parolia A., Qiao Y., Bawa P., Eyunni S., Mannan R., Carson S.E., Chang Y., Wang X., Zhang Y. et al. Targeting SWI/SNF ATPases in enhancer-addicted prostate cancer. Nature. 2022; 601:434–439. 34937944 10.1038/s41586-021-04246-z PMC8770127

[18] Hoffman J.A., Trotter K.W., Ward J.M., Archer T.K. BRG1 governs glucocorticoid receptor interactions with chromatin and pioneer factors across the genome. eLife. 2018; 7:e35073. 29792595 10.7554/eLife.35073 PMC5967868

[19] Frederick M.A., Williamson K.E., Fernandez Garcia M., Ferretti M.B., McCarthy R.L., Donahue G., Luzete Monteiro E., Takenaka N., Reynaga J., Kadoch C. et al. A pioneer factor locally opens compacted chromatin to enable targeted ATP-dependent nucleosome remodeling. Nat. Struct. Mol. Biol. 2023; 30:31–37. 36536103 10.1038/s41594-022-00886-5 PMC10004348

[20] Pan J., McKenzie Z.M., D’Avino A.R., Mashtalir N., Lareau C.A., St Pierre R., Wang L., Shilatifard A., Kadoch C. The ATPase module of mammalian SWI/SNF family complexes mediates subcomplex identity and catalytic activity-independent genomic targeting. Nat. Genet. 2019; 51:618–626. 30858614 10.1038/s41588-019-0363-5 PMC6755913

[21] Lai A.Y., Wade P.A. Cancer biology and NuRD: a multifaceted chromatin remodelling complex. Nat. Rev. Cancer. 2011; 11:588–596. 21734722 10.1038/nrc3091 PMC4157524

[22] O'Shaughnessy A., Hendrich B. CHD4 in the DNA-damage response and cell cycle progression: not so NuRDy now. Biochem. Soc. Trans. 2013; 41:777–782. 23697937 10.1042/BST20130027 PMC3685327

[23] Wilczewski C.M., Hepperla A.J., Shimbo T., Wasson L., Robbe Z.L., Davis I.J., Wade P.A., Conlon F.L. CHD4 and the NuRD complex directly control cardiac sarcomere formation. Proc. Natl. Acad. Sci. U.S.A. 2018; 115:6727–6732. 29891665 10.1073/pnas.1722219115 PMC6042121

[24] Weiss K., Lazar H.P., Kurolap A., Martinez A.F., Paperna T., Cohen L., Smeland M.F., Whalen S., Heide S., Keren B. et al. Correction: the CHD4-related syndrome: a comprehensive investigation of the clinical spectrum, genotype-phenotype correlations, and molecular basis. Genet. Med. 2020; 22:669. 31844176 10.1038/s41436-019-0727-3

[25] Nitarska J., Smith J.G., Sherlock W.T., Hillege M.M., Nott A., Barshop W.D., Vashisht A.A., Wohlschlegel J.A., Mitter R., Riccio A. A functional switch of NuRD chromatin remodeling complex subunits regulates mouse cortical development. Cell Rep. 2016; 17:1683–1698. 27806305 10.1016/j.celrep.2016.10.022 PMC5149529

[26] Xia L., Huang W., Bellani M., Seidman M.M., Wu K., Fan D., Nie Y., Cai Y., Zhang Y.W., Yu L.R. et al. CHD4 Has oncogenic functions in initiating and maintaining epigenetic suppression of multiple tumor suppressor genes. Cancer Cell. 2017; 31:653–668. 28486105 10.1016/j.ccell.2017.04.005 PMC5587180

[27] Zhao S., Choi M., Overton J.D., Bellone S., Roque D.M., Cocco E., Guzzo F., English D.P., Varughese J., Gasparrini S. et al. Landscape of somatic single-nucleotide and copy-number mutations in uterine serous carcinoma. Proc. Natl. Acad. Sci. U.S.A. 2013; 110:2916–2921. 23359684 10.1073/pnas.1222577110 PMC3581983

[28] Novillo A., Fernández-Santander A., Gaibar M., Galán M., Romero-Lorca A., El Abdellaoui-Soussi F., Gómez-Del Arco P. Role of chromodomain-helicase-DNA-binding protein 4 (CHD4) in breast cancer. Front. Oncol. 2021; 11:633233. 33981601 10.3389/fonc.2021.633233 PMC8107472

[29] Si W., Huang W., Zheng Y., Yang Y., Liu X., Shan L., Zhou X., Wang Y., Su D., Gao J. et al. Dysfunction of the reciprocal feedback loop between GATA3- and ZEB2-nucleated repression programs contributes to breast cancer metastasis. Cancer Cell. 2015; 27:822–836. 26028330 10.1016/j.ccell.2015.04.011

[30] Luo C.W., Wu C.C., Chang S.J., Chang T.M., Chen T.Y., Chai C.Y., Chang C.L., Hou M.F., Pan M.R. CHD4-mediated loss of E-cadherin determines metastatic ability in triple-negative breast cancer cells. Exp. Cell. Res. 2018; 363:65–72. 29305962 10.1016/j.yexcr.2017.12.032

[31] Wang Y., Chen Y., Bao L., Zhang B., Wang J.E., Kumar A., Xing C., Wang Y., Luo W. CHD4 Promotes breast cancer progression as a coactivator of hypoxia-inducible factors. Cancer Res. 2020; 80:3880–3891. 32699137 10.1158/0008-5472.CAN-20-1049 PMC7501193

[32] Burgold T., Barber M., Kloet S., Cramard J., Gharbi S., Floyd R., Kinoshita M., Ralser M., Vermeulen M., Reynolds N. et al. The nucleosome Remodelling and deacetylation complex suppresses transcriptional noise during lineage commitment. EMBO J. 2019; 38:e100788. 31036553 10.15252/embj.2018100788 PMC6576150

[33] Robbe Z.L., Shi W., Wasson L.K., Scialdone A.P., Wilczewski C.M., Sheng X., Hepperla A.J., Akerberg B.N., Pu W.T., Cristea I.M. et al. CHD4 is recruited by GATA4 and NKX2-5 to repress noncardiac gene programs in the developing heart. Genes Dev. 2022; 36:468–482. 35450884 10.1101/gad.349154.121 PMC9067406

[34] Reynolds N., Latos P., Hynes-Allen A., Loos R., Leaford D., O'Shaughnessy A., Mosaku O., Signolet J., Brennecke P., Kalkan T. et al. NuRD suppresses pluripotency gene expression to promote transcriptional heterogeneity and lineage commitment. Cell Stem Cell. 2012; 10:583–594. 22560079 10.1016/j.stem.2012.02.020 PMC3402183

[35] Sims J.K., Wade P.A. Mi-2/NuRD complex function is required for normal S phase progression and assembly of pericentric heterochromatin. Mol. Biol. Cell. 2011; 22:3094–3102. 21737684 10.1091/mbc.E11-03-0258 PMC3164457

[36] Shimbo T., Du Y., Grimm S.A., Dhasarathy A., Mav D., Shah R.R., Shi H., Wade P.A. MBD3 localizes at promoters, gene bodies and enhancers of active genes. PLoS Genet. 2013; 9:e1004028. 24385926 10.1371/journal.pgen.1004028 PMC3873231

[37] Yoshida T., Hazan I., Zhang J., Ng S.Y., Naito T., Snippert H.J., Heller E.J., Qi X., Lawton L.N., Williams C.J. et al. The role of the chromatin remodeler mi-2beta in hematopoietic stem cell self-renewal and multilineage differentiation. Genes Dev. 2008; 22:1174–1189. 18451107 10.1101/gad.1642808 PMC2335314

[38] Williams C.J., Naito T., Arco P.G., Seavitt J.R., Cashman S.M., De Souza B., Qi X., Keables P., Von Andrian U.H., Georgopoulos K. The chromatin remodeler mi-2beta is required for CD4 expression and T cell development. Immunity. 2004; 20:719–733. 15189737 10.1016/j.immuni.2004.05.005

[39] Morris S.A., Baek S., Sung M.H., John S., Wiench M., Johnson T.A., Schiltz R.L., Hager G.L. Overlapping chromatin-remodeling systems collaborate genome wide at dynamic chromatin transitions. Nat. Struct. Mol. Biol. 2014; 21:73–81. 24317492 10.1038/nsmb.2718 PMC3947387

[40] Takaku M., Grimm S.A., Shimbo T., Perera L., Menafra R., Stunnenberg H.G., Archer T.K., Machida S., Kurumizaka H., Wade P.A. GATA3-dependent cellular reprogramming requires activation-domain dependent recruitment of a chromatin remodeler. Genome Biol. 2016; 17:36. 26922637 10.1186/s13059-016-0897-0 PMC4769547

[41] Takaku M., Grimm S.A., Roberts J.D., Chrysovergis K., Bennett B.D., Myers P., Perera L., Tucker C.J., Perou C.M., Wade P.A. GATA3 zinc finger 2 mutations reprogram the breast cancer transcriptional network. Nat. Commun. 2018; 9:1059. 29535312 10.1038/s41467-018-03478-4 PMC5849768

[42] Heinz S., Benner C., Spann N., Bertolino E., Lin Y.C., Laslo P., Cheng J.X., Murre C., Singh H., Glass C.K. Simple combinations of lineage-determining transcription factors prime cis-regulatory elements required for macrophage and B cell identities. Mol. Cell. 2010; 38:576–589. 20513432 10.1016/j.molcel.2010.05.004 PMC2898526

[43] McCarthy M.T., O’Callaghan C.A. PeaKDEck: a kernel density estimator-based peak calling program for DNaseI-seq data. Bioinformatics. 2014; 30:1302–1304. 24407222 10.1093/bioinformatics/btt774 PMC3998130

[44] Varet H., Brillet-Guéguen L., Coppée J.Y., Dillies M.A. SARTools: a DESeq2- and EdgeR-based R pipeline for comprehensive differential analysis of RNA-seq data. PLoS One. 2016; 11:e0157022. 27280887 10.1371/journal.pone.0157022 PMC4900645

[45] Robinson M.D., McCarthy D.J., Smyth G.K. edgeR: a bioconductor package for differential expression analysis of digital gene expression data. Bioinformatics. 2010; 26:139–140. 19910308 10.1093/bioinformatics/btp616 PMC2796818

[46] Cooper M., Ray A., Bhattacharya A., Dhasarathy A., Takaku M. ATAC-seq optimization for Cancer Epigenetics research. J. Vis. Exp. 2022; 184:e64242. 10.3791/64242 PMC10064075 35848835

[47] Langmead B., Trapnell C., Pop M., Salzberg S.L. Ultrafast and memory-efficient alignment of short DNA sequences to the human genome. Genome Biol. 2009; 10:R25. 19261174 10.1186/gb-2009-10-3-r25 PMC2690996

[48] Tanaka H., Takizawa Y., Takaku M., Kato D., Kumagawa Y., Grimm S.A., Wade P.A., Kurumizaka H. Interaction of the pioneer transcription factor GATA3 with nucleosomes. Nat. Commun. 2020; 11:4136. 32811816 10.1038/s41467-020-17959-y PMC7434886

[49] Dobin A., Davis C.A., Schlesinger F., Drenkow J., Zaleski C., Jha S., Batut P., Chaisson M., Gingeras T.R. STAR: ultrafast universal RNA-seq aligner. Bioinformatics. 2013; 29:15–21. 23104886 10.1093/bioinformatics/bts635 PMC3530905

[50] Love M.I., Huber W., Anders S. Moderated estimation of fold change and dispersion for RNA-seq data with DESeq2. Genome Biol. 2014; 15:550. 25516281 10.1186/s13059-014-0550-8 PMC4302049

[51] Huang da W., Sherman B.T., Lempicki R.A. Systematic and integrative analysis of large gene lists using DAVID bioinformatics resources. Nat. Protoc. 2009; 4:44–57. 19131956 10.1038/nprot.2008.211

[52] Huang da W., Sherman B.T., Lempicki R.A. Bioinformatics enrichment tools: paths toward the comprehensive functional analysis of large gene lists. Nucleic Acids Res. 2009; 37:1–13. 19033363 10.1093/nar/gkn923 PMC2615629

[53] Adomas A.B., Grimm S.A., Malone C., Takaku M., Sims J.K., Wade P.A. Breast tumor specific mutation in GATA3 affects physiological mechanisms regulating transcription factor turnover. BMC Cancer. 2014; 14:278. 24758297 10.1186/1471-2407-14-278 PMC4021073

[54] Buenrostro J.D., Giresi P.G., Zaba L.C., Chang H.Y., Greenleaf W.J. Transposition of native chromatin for fast and sensitive epigenomic profiling of open chromatin, DNA-binding proteins and nucleosome position. Nat. Methods. 2013; 10:1213–1218. 24097267 10.1038/nmeth.2688 PMC3959825

[55] Yan W., Cao Q.J., Arenas R.B., Bentley B., Shao R. GATA3 inhibits breast cancer metastasis through the reversal of epithelial-mesenchymal transition. J. Biol. Chem. 2010; 285:14042–14051. 20189993 10.1074/jbc.M110.105262 PMC2859565

[56] Takaku M., Grimm S.A., De Kumar B., Bennett B.D., Wade P.A. Cancer-specific mutation of GATA3 disrupts the transcriptional regulatory network governed by estrogen receptor alpha, FOXA1 and GATA3. Nucleic Acids Res. 2020; 48:4756–4768. 32232341 10.1093/nar/gkaa179 PMC7229857

[57] Home P., Kumar R.P., Ganguly A., Saha B., Milano-Foster J., Bhattacharya B., Ray S., Gunewardena S., Paul A., Camper S.A. et al. Genetic redundancy of GATA factors in the extraembryonic trophoblast lineage ensures the progression of preimplantation and postimplantation mammalian development. Development. 2017; 144:876–888. 28232602 10.1242/dev.145318 PMC5374352

[58] Ralston A., Cox B.J., Nishioka N., Sasaki H., Chea E., Rugg-Gunn P., Guo G., Robson P., Draper J.S., Rossant J. Gata3 regulates trophoblast development downstream of Tead4 and in parallel to Cdx2. Development. 2010; 137:395–403. 20081188 10.1242/dev.038828

[59] Li B., Carey M., Workman J.L. The role of chromatin during transcription. Cell. 2007; 128:707–719. 17320508 10.1016/j.cell.2007.01.015

[60] Zhu F., Farnung L., Kaasinen E., Sahu B., Yin Y., Wei B., Dodonova S.O., Nitta K.R., Morgunova E., Taipale M. et al. The interaction landscape between transcription factors and the nucleosome. Nature. 2018; 562:76–81. 30250250 10.1038/s41586-018-0549-5 PMC6173309

[61] Iwafuchi-Doi M., Donahue G., Kakumanu A., Watts J.A., Mahony S., Pugh B.F., Lee D., Kaestner K.H., Zaret K.S. The pioneer transcription factor FoxA maintains an accessible nucleosome configuration at enhancers for tissue-specific gene activation. Mol. Cell. 2016; 62:79–91. 27058788 10.1016/j.molcel.2016.03.001 PMC4826471

[62] Chen Y., Bates D.L., Dey R., Chen P.H., Machado A.C., Laird-Offringa I.A., Rohs R., Chen L. DNA binding by GATA transcription factor suggests mechanisms of DNA looping and long-range gene regulation. Cell Rep. 2012; 2:1197–1206. 23142663 10.1016/j.celrep.2012.10.012 PMC3978094

[63] Dodonova S.O., Zhu F., Dienemann C., Taipale J., Cramer P. Nucleosome-bound SOX2 and SOX11 structures elucidate pioneer factor function. Nature. 2020; 580:669–672. 32350470 10.1038/s41586-020-2195-y

[64] Michael A.K., Grand R.S., Isbel L., Cavadini S., Kozicka Z., Kempf G., Bunker R.D., Schenk A.D., Graff-Meyer A., Pathare G.R. et al. Mechanisms of OCT4-SOX2 motif readout on nucleosomes. Science. 2020; 368:1460–1465. 32327602 10.1126/science.abb0074

[65] Zaret K.S., Mango S.E. Pioneer transcription factors, chromatin dynamics, and cell fate control. Curr. Opin. Genet. Dev. 2016; 37:76–81. 26826681 10.1016/j.gde.2015.12.003 PMC4914445

[66] Luo M., Ling T., Xie W., Sun H., Zhou Y., Zhu Q., Shen M., Zong L., Lyu G., Zhao Y. et al. NuRD blocks reprogramming of mouse somatic cells into pluripotent stem cells. Stem Cells. 2013; 31:1278–1286. 23533168 10.1002/stem.1374

[67] Rais Y., Zviran A., Geula S., Gafni O., Chomsky E., Viukov S., Mansour A.A., Caspi I., Krupalnik V., Zerbib M. et al. Deterministic direct reprogramming of somatic cells to pluripotency. Nature. 2013; 502:65–70. 24048479 10.1038/nature12587

[68] Whyte W.A., Bilodeau S., Orlando D.A., Hoke H.A., Frampton G.M., Foster C.T., Cowley S.M., Young R.A. Enhancer decommissioning by LSD1 during embryonic stem cell differentiation. Nature. 2012; 482:221–225. 22297846 10.1038/nature10805 PMC4144424

[69] Reynolds N., Salmon-Divon M., Dvinge H., Hynes-Allen A., Balasooriya G., Leaford D., Behrens A., Bertone P., Hendrich B. NuRD-mediated deacetylation of H3K27 facilitates recruitment of Polycomb Repressive Complex 2 to direct gene repression. EMBO J. 2012; 31:593–605. 22139358 10.1038/emboj.2011.431 PMC3273378

[70] Bornelöv S., Reynolds N., Xenophontos M., Gharbi S., Johnstone E., Floyd R., Ralser M., Signolet J., Loos R., Dietmann S. et al. The nucleosome remodeling and deacetylation complex modulates chromatin structure at sites of active transcription to fine-tune gene expression. Mol. Cell. 2018; 71:56–72. 30008319 10.1016/j.molcel.2018.06.003 PMC6039721

[71] Blossey R., Schiessel H. Kinetic proofreading of gene activation by chromatin remodeling. Hfsp j. 2008; 2:167–170. 19404470 10.2976/1.2909080 PMC2645567

[72] Schiessel H., Blossey R. Pioneer transcription factors in chromatin remodeling: the kinetic proofreading view. Phys. Rev. E. 2020; 101:040401. 32422793 10.1103/PhysRevE.101.040401

