# Supplementary material for: Genomic transcription factor binding site selection is edited by the chromatin remodeling factor CHD4
Source: Nucleic Acids Res. 2024 Jan 28;52(7):3607–22. doi: 10.1093/nar/gkae025 (PMC11039999; doi:10.1093/nar/gkae025)
Supplement: gkae025_Supplemental_Files [file gkae025_supplemental_files.zip › Supplementary_Figures.pdf]

Saotome et al. Supplementary Figure 1

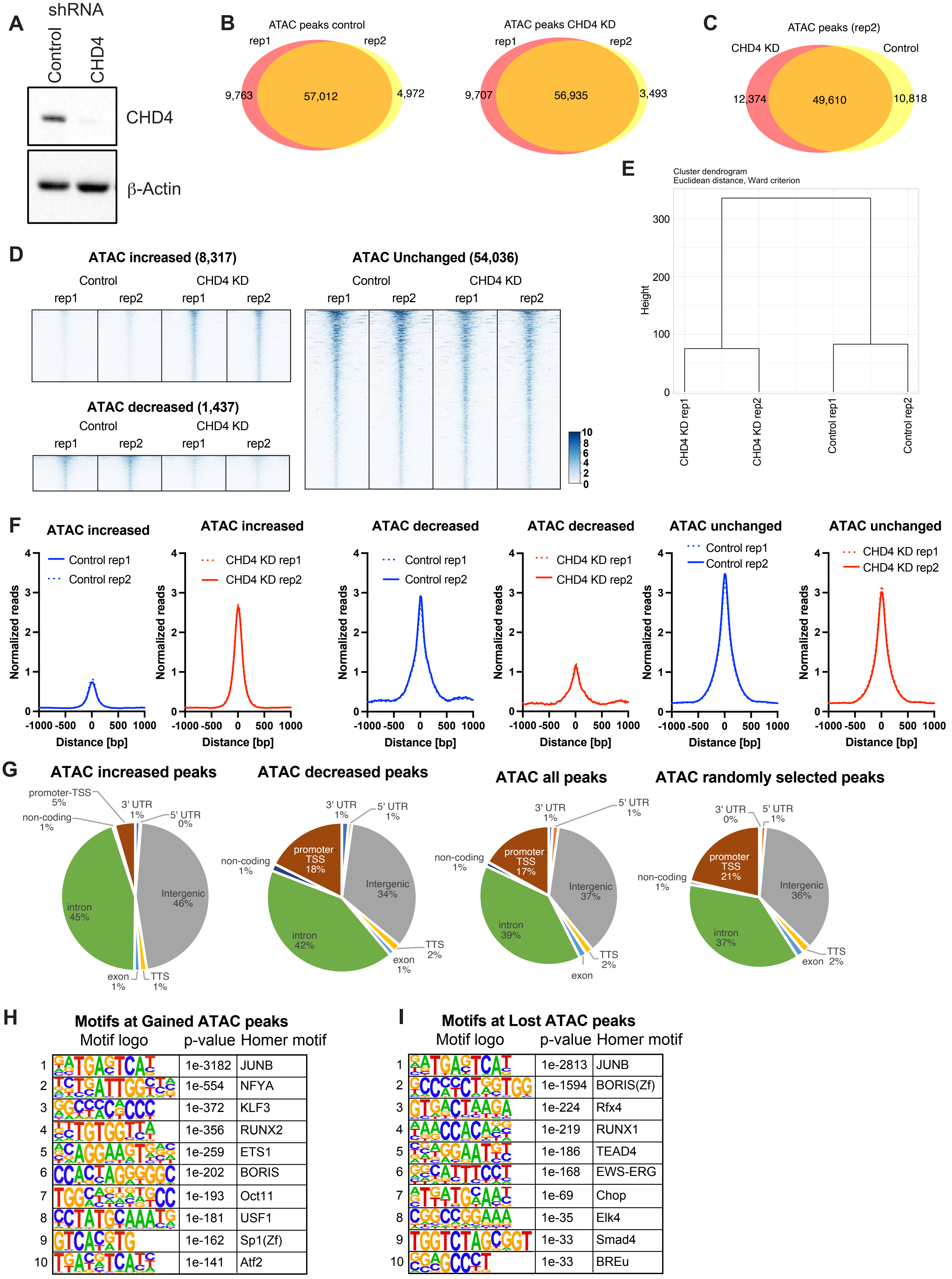

Supplementary Figure 1. Impact of CHD4 knockdown on chromatin accessibility
(A) Western blot showing CHD4 knockdown. MDA-MB-231 cells were infected with the lentivirus encoding control shRNA or CHD4 shRNA. β-Actin expression was used as an internal control. **(B)** Venn diagram showing the ATAC-seq peak overlap between biological replicates in control (top) or CHD4 knockdown (KD, bottom) cells. **(C)** Venn diagram showing the ATAC-seq peak overlap between control and CHD4 knockdown MDA-MB-231 cells. **(D)** Heatmap showing the reproducibility of ATAC-seq signals within biological replicates at increased, decreased, or unchanged ATAC-seq peaks. **(E)** Hierarchical clustering of ATAC-seq data was performed, with the dendrogram generated using Counts Per Million (CPM) data. We computed an Euclidean distance between samples and constructed the dendrogram based on the Ward criterion. Consistent with the heatmap results, biological replicates clustered together, further confirming the reproducibility of our ATAC-seq data. **(F)** Metaplot displaying normalized ATAC-seq reads per peak from two biological replicates, categorized at differentially expressed (either increased or decreased) or unchanged peaks. **(G)** Pie chart showing peak annotation defined by HOMER. Increased, decreased, all, or randomly selected ATAC-seq peaks are classified into 8 annotation categories. **(H-I)** HOMER de novo motif analysis. ATAC-seq peaks are grouped in Gained (E, uniquely observed in CHD4 knockdown cells) or Lost (F, only observed in the control cells) ATAC-seq peaks (shown in Figure 1B), and are used as input for HOMER motif analysis.

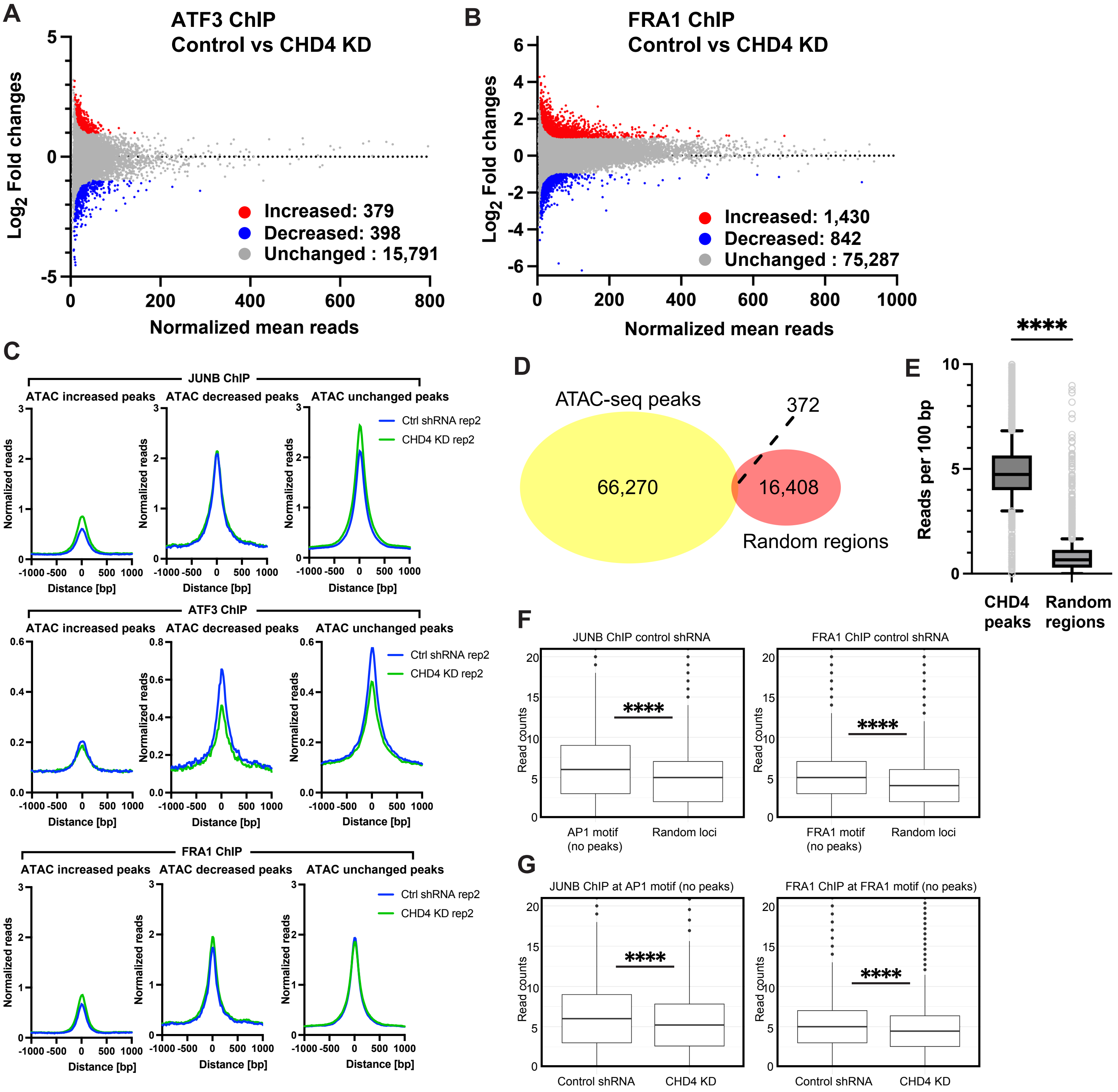

Supplementary Figure 2. Differential peak analyses of ATF3 and FRA1 ChIP-seq data.
(A) Scatter plot shows increased (red), decreased (blue), and unchanged (grey) ATF3 peaks upon CHD4 depletion. **(B)** Scatter plot shows increased (red), decreased (blue), and unchanged (grey) FRA1 peaks upon CHD4 depletion. **(C)** Metaplot showing normalized ChIP-seq reads/peak at ATAC-seq differential peaks, derived from the second biological replicate. JUNB (top). ATF3 (middle), and FRA1 (bottom) signals in control (blue) or CHD4 KD cells (green) are plotted. **(D)** Venn diagram shows the overlap between ATAC-seq peaks and randomly selected genomic regions. Random regions were selected by shuffleBed (bedtools), and any overlap with CHD4 peaks was avoided. **(E)** Box plot displays normalized reads at CHD4 peaks (left) and at randomly selected loci (right). **(F)** Quantitative analysis of JUNB or FRA1 ChIP-seq data shown in Figure 3B and 3C. Reads are collected between -500 bp to +500 bp at AP1 motif cotaining or randomly selected loci. **(G)** Quantitative analysis of JUNB or FRA1 ChIP-seq shown in Figure 3D and 3E. The same window (from -500 bp to +500 bp) was used to measure read counts in control or CHD4 KD cells. **** $p < 0.0001$.

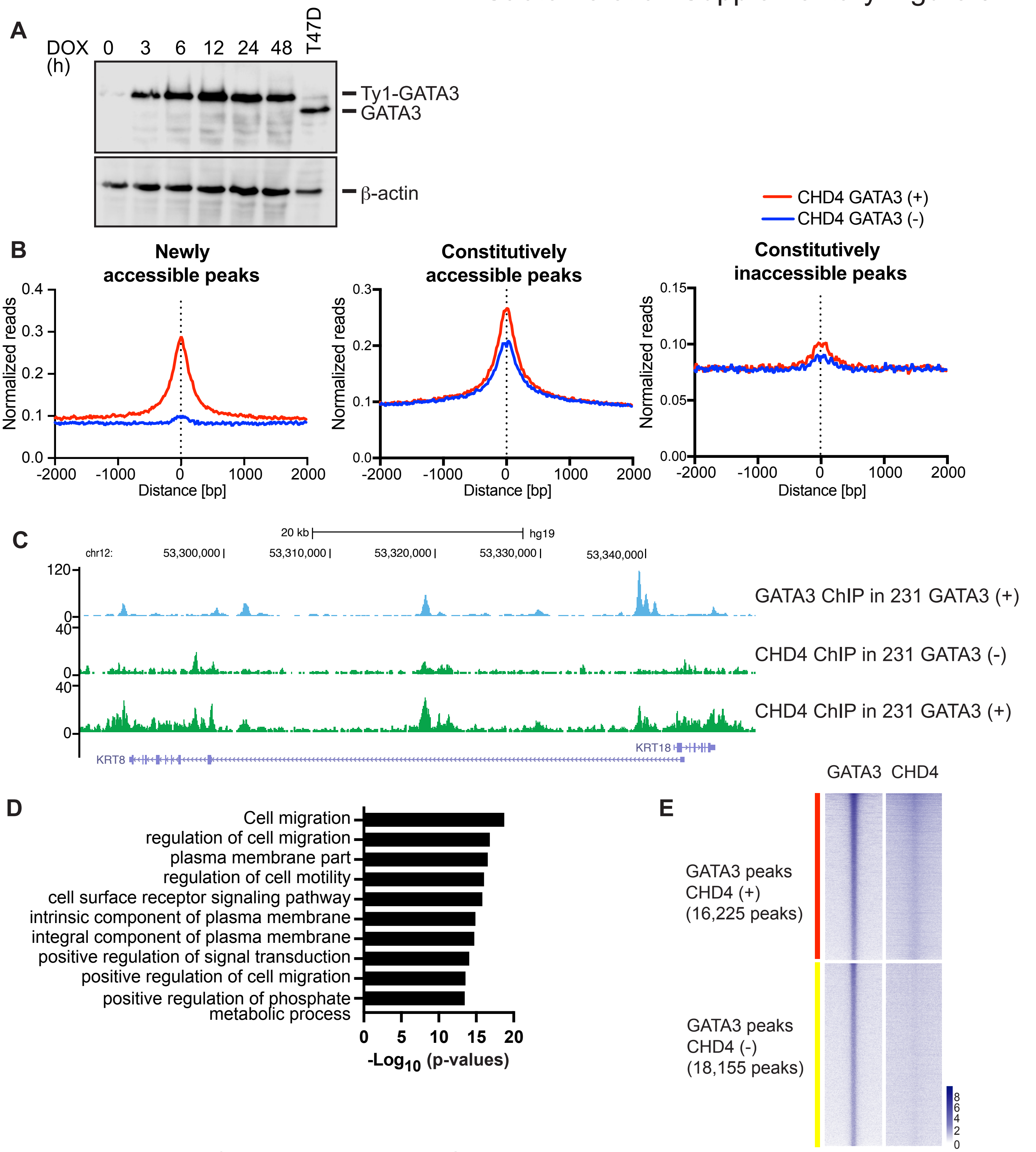

Supplementary Figure 3. CHD4 colocalizes with GATA3 in MET.
(A) Western blot showing GATA3 expression after DOX treatment (1 μg/ml at the final concentration). β-Actin expression was used as an internal control. **(B)** Metaplots showing the CHD4 ChIP-seq signals before and after GATA3 expression. Newly accessible, constitutively accessible. constitutively inaccessible peak groups are previously defined in the GATA3 stably expressed cell system 25. **(C)** Representative genome track of GATA3 and CHD4 ChIP-seq data. GATA3 ChIP-seq was performed in the GATA3-expressed stable cell line. CHD4 ChIP-seq was performed in the GATA3 negative or positive MDA-MB-231 stable cell lines. **(D)** Pathway analysis of the up-regulated genes shown in Figure 4A. Top 10 enriched pathways are shown. expression system. **(E)** Heatmap displays the colocalization of GATA3 and CHD4 in T47D cells. GATA3 ChIP-seq peaks are classified as CHD4 positive or negative based on the presence of CHD4 ChIP-seq signals compared to the background level.

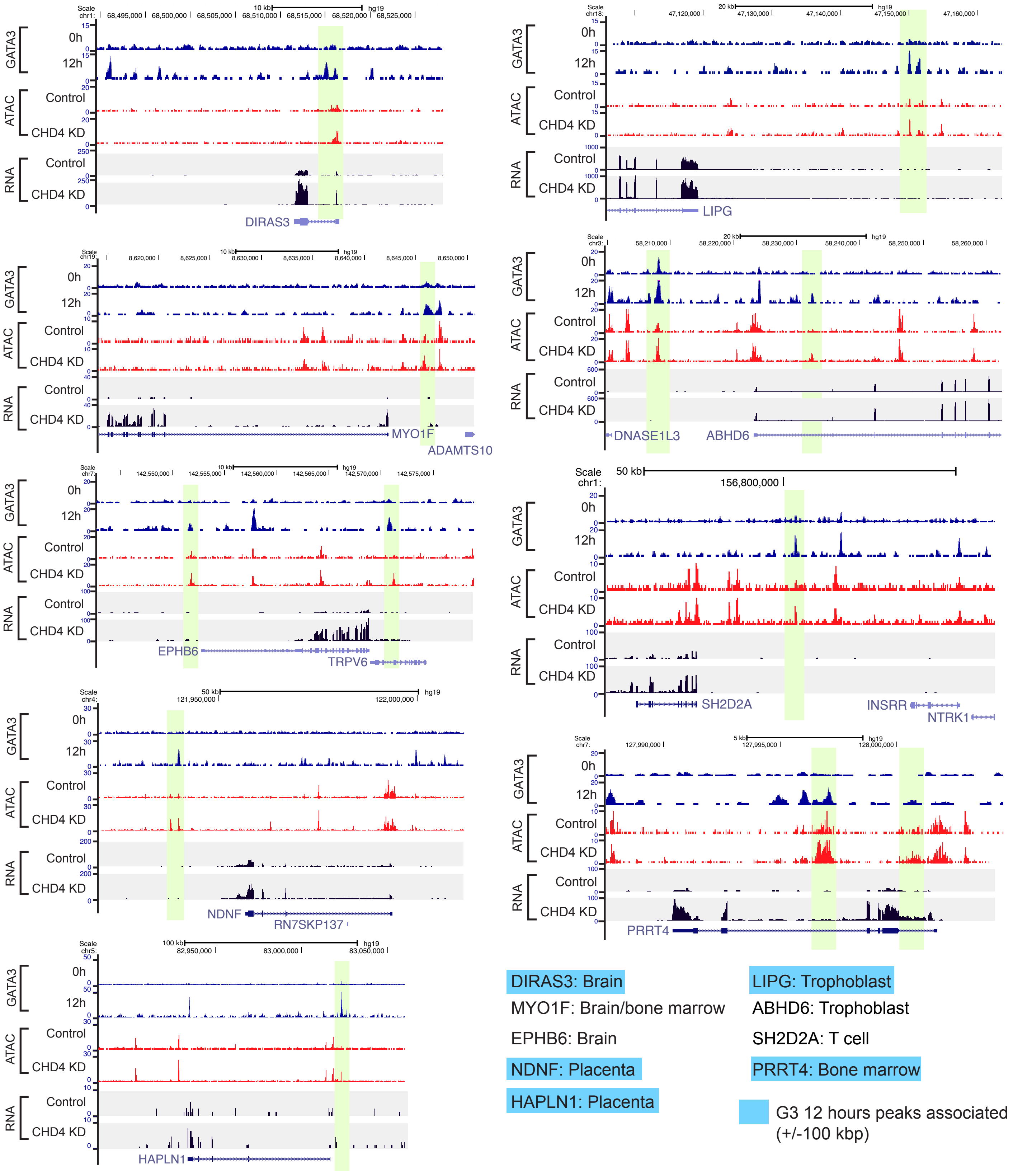

Supplementary Figure 4. CHD4 depletion induces aberrant chromatin opening and gene activation. Genome browser tracks show the examples of aberrant gene expression. Brain, placenta, trophoblast, T cell, and bone marrow related genes are selected. In each figure, GATA3 peaks that have increased ATAC-seq signals in the CHD4 knockdown cells are highlighted. The genes that are associated with the constitutively inaccessible GATA3 peaks (peaks within +/- 100 kbp) were highlighted in light blue.

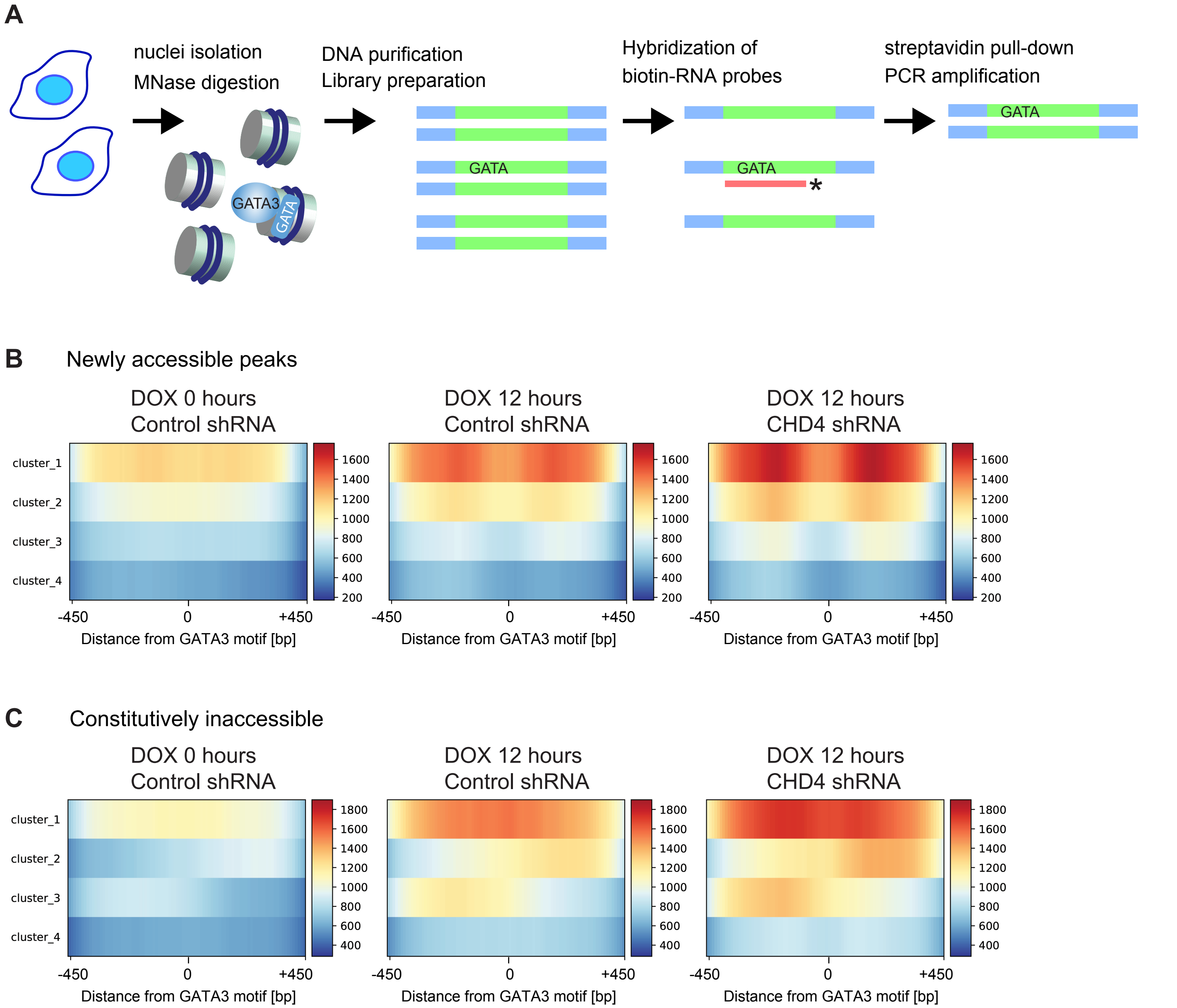

Supplementary Figure 5. Capture MNase-seq
(A) Experimental scheme of capture MNase-seq. Mono-nucleosmal fragments were prepared by MNase digestion. Sequencing libraries were made by NEXTFLEX Rapid DNA-Seq kit (PerkinElmer). Biotinylated RNA probes (Agilent) were used to enrich nucleosome fragments at selected GATA3 peaks. **(B)** Heatmap shows nucleosome density (MNase-seq fragment coverage) at newly accessible peaks. CapMNase-seq data were normalized to 20 million reads. **(C)** Heatmap shows nucleosome density at constitutively inaccessible peaks.
